# Reduction in suicides and suicide attempts following implementation of AI-based video surveillance in the Stockholm metro system: an intervention study

**DOI:** 10.1186/s12889-026-28358-1

**Published:** 2026-07-01

**Authors:** Johan Fredin-Knutzén, Gergö Hadlaczky, Anna-Lena Andersson, Marcus Sokolowski

**Affiliations:** 1 National Centre for Suicide Research and Prevention of Mental Ill-Health (NASP), Karolinska Institutet, Stockholm, S-171 77 Sweden; 2 CHIS, Stockholm Health Care Services (SLSO), Region Stockholm, Stockholm, Sweden

**Keywords:** Suicide, Metro, Subway, Station, Railway, Closed-circuit television (CCTV), Artificial Intelligence (AI)

## Abstract

**Background:**

Over 700,000 suicides occurring globally each year are a major public health issue. Railways and metros provide a lethal means of suicide, mainly occurring by persons under train (PUT) events. Restriction of means has been shown to be effective in reducing suicides and is increasingly being prioritized in railway settings, e.g., using physical barriers. Here, we instead investigated the changes in suicidal behavior on metro platforms following the implementation of an AI-based CCTV detection system.

**Methods:**

We used longitudinal data about PUT due to suicidality in the Swedish metro system in Stockholm (2010–2025). A controlled interrupted time series (CITS) analysis, as well as uncontrolled analyses, were used to test if an AI-CCTV implementation in Q4 2021 (at 14 stations) was associated with decreased rates of PUT due to suicidality, compared to the other 86 stations as controls. We also evaluated secondary outcomes (e.g., suicide deaths and train-traffic cancellations). Sensitivity analyses assessed the robustness of the primary model. A separate exploratory analysis examined an extended post-period, which included multiple heterogeneous system-wide exposures.

**Results:**

Rates of PUT due to suicidality were lower after AI-CCTV implementation, in analyses with (*IRR* = 0.27, *p* < 0.05) or without (*IRR* = 0.41, *p* < 0.05) controls. Secondary outcomes showed consistent point estimates, e.g. death by suicide after PUT (*IRR* = 0.3), safeguarded individuals (*IRR* = 0.8) and less cancelled train-kilometers in the metro system. The robustness of the changes in PUT and death by suicide outcomes were confirmed by Bayesian sensitivity analyses using weak priors.

**Conclusions:**

Implementation of AI-CCTV as described herein was associated with lower rates of PUT due to suicidality at metro stations, as well as changes in the same direction for e.g. deaths by suicide and less cancelled train-kilometers. This preliminary study of AI-CCTV in the metro system provides a specific example of how such an intervention may support suicide prevention in the metro system, at least in the short-term.

**Supplementary Information:**

The online version contains supplementary material available at 10.1186/s12889-026-28358-1.

## Introduction

Over 700,000 suicide deaths and 4–14 times as many suicide attempts occur globally each year, representing a leading cause of death in people aged under 50 and an urgent public health concern [1, 2]. Railways and metros provide a highly lethal means of suicide in the public setting. Suicide is the most common cause of death on railways. For the EU railways, the annual number of suicides stayed between roughly 2,200 and 2,800 from 2010 to 2023, while 841 fatalities from other causes occurred on EU railways in 2023 [3]. These numbers do not include deaths in the metro systems within the EU, where additional suicide and accident deaths occur each year. The number of suicides *vs* accidents in rail and metro systems is most likely underestimated; evidence from Sweden shows that more thorough investigations of lethal events further increased the number of deaths classified as suicides [4]. Injury events whereby a person is struck by a train, referred to as persons under train (PUT), represent severe and often fatal outcomes. In addition, such events have substantial consequences for train drivers [5], passengers, and the transport system, including psychological distress, service disruptions, and socioeconomic costs.

Platform screen doors are the single measure with the most significant evidence of preventing suicides in station areas [6]. This is one of so-called means restriction interventions, which are generally recommended approaches for suicide prevention [7]. The downside of such a system is that it is often complex and costly to install, thereby less likely to become used more broadly. Globally, the vast majority of rail and metro stations therefore lack platform screen doors. Another preventive measure which has been suggested for a long time is using closed-circuit television (CCTV) cameras for suicide prevention in rail environments; it was proposed as early as 1994 as a potential measure for preventing suicides in the London underground [8]. More recently, ideas about using artificial intelligence (AI) to scan the CCTV video feeds for suicidal behavior has become a prioritized area within the rail industry. Such AI-CCTV systems aim to enable early identification of an elevated suicide risk and rapid operational responses to mitigate the risk of a suicide in front of a train in station areas. In late 2024, the International Union of Railways (UIC) and five of Europe’s largest infrastructure managers launched the AI4SAFEBEHAVE project, reflecting the growing attention to using AI to monitor human behavior and prevent suicide and trespassing [9]. A scoping review of suicide prevention measures at railroads published in 2024, identified 26 studies that empirically evaluated suicide prevention measures in a rail-related context [6]. However, despite growing implementation in practice, there is to our knowledge no published evidence about whether AI-CCTV actually reduces suicides and suicide attempts in real-world rail or metro systems, highlighting a need for evaluation focused on suicidal outcomes per se using controlled or quasi-experimental studies. Furthermore, a recent systematic review concluded that any putative benefits of using surveillance technologies for suicide prevention in public spaces, requires more evidence [10].

The primary objective of this study was to evaluate whether AI-CCTV was associated with a reduction in the incidence of PUT due to suicidality (suicide and suicide attempts) in the Swedish metro system, located in the main capital city of Stockholm. This outcome reflects the intended purpose for installing the AI-CCTV, i.e. preventing events which result in death by suicide or suicide attempts with a strong intent to die, given the expected high lethality of the method. Influence of the AI-CCTV on rates of PUT due to suicidality was studied by using controlled and uncontrolled, longitudinal pre–post quasi-experimental time series designs [11–13]. A key secondary objective was to assess the impact of AI-CCTV on the number of suicide deaths due to PUT, defined as the fatal subset of PUT due to suicidality. Additional secondary objectives included changes on deaths by suicide due to PUT, as well as other operational and safety-related outcomes, such as putative suicide attempts interrupted before track entry, PUT due to other reasons than suicidality and train cancellations related to trespassing and PUT.

## Methods

### Study design

This study adheres to the STROBE statement for observational cohort studies [14] (Table S3 in Additional File 1). We conducted a longitudinal study about PUT due to suicidality from year 2010 to 2025, as well as about other secondary outcomes and sub-periods (Table 1), using controlled and uncontrolled, quasi-experimental pre-post time series design [11–13, 15] with AI-CCTV intervention as exposure.

**Table 1:** Timing of study outcomes, exposure, and concurrent external events

Variable	Start (inclusive)	End (inclusive)
*Primary outcome*		
PUT due to suicidality (suicides and suicide attempts)	Q1 2010	Q3 2022
*Key Secondary outcome*		
Death by suicide due to PUT	Q1 2010	Q3 2022
*Secondary outcomes*		
Individuals safeguarded for suicide risk	Q1 2018	Q3 2022
Canceled train services	Q1 2011	Q3 2022
PUT due to accident	Q1 2010	Q3 2022
*Exposure*		
AI-CCTV, primary post-period	Q4 2021	Q3 2022
AI-CCTV, extended post-period^a^	Q4 2022	Q2 2025^a^
*Concurrent external events (potential history bias)*		
Extensive media coverage of the intervention	Q4 2022	Q2 2025^a^

^a^Data from Q4 2022 and onward were used for exploratory analyses and thus not included in the primary analysis

### Intervention exposure

**Fig. 1:**
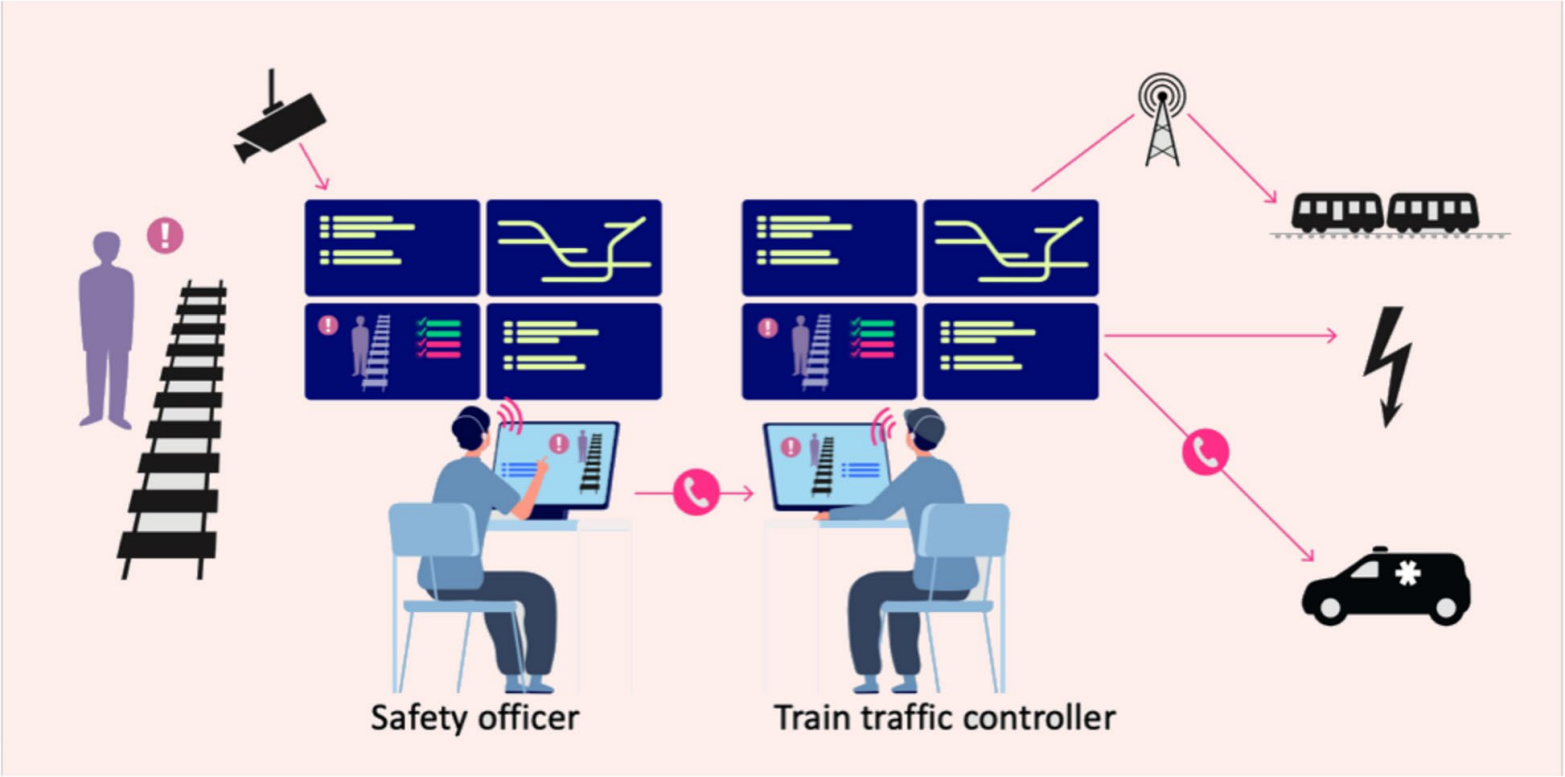
Overview of the AI-CCTV intervention. The AI-CCTV scans the feed from cameras on the platforms. An alarm is triggered when a predefined behaviour is recognized. A safety officer manually reviews the alarm. Events which assessed as having elevated risk for PUT are transferred to a train traffic controller, who intervenes by warning the train driver, turning off the electricity, and calling for emergency services

The AI-CCTV system was implemented at 14 of 100 stations in the Stockholm metro in October 2021 by the regional public transport authority (Storstockholms Lokaltrafik, SL). SL selected these 14 stations because they were identified as more affected by trespassing. The AI-CCTV automatically scans the video feeds from existing CCTV cameras. Upon detection, an alarm is triggered and a safety officer manually reviews and assesses the risk of a PUT. When the safety officer confirms the elevated risk, various actions are taken immediately to mitigate the risk for PUT (Fig. 1). Although the AI-CCTV system could prevent a large number of PUT events, it cannot prevent events where the risk behaviors appear immediately before a train arrives, due to insufficient time for handling the alarm and braking to avoid the person-train collision. Initially, the AI-CCTV system included features that detected when a person entered the track area (trespassing) or stood immediately at the platform edge. Additional exposures occurred during an extended post-period, from the fourth quarter (Q4) 2022 onward. More details are presented in the Additional file 1.

### Outcome measures

The primary outcome of all injury events occurring on the Stockholm Metro was retrieved from the Swedish Transport Agency (STA) TRAP register database. The primary outcome included injury events from a PUT or contact with the electric rail, as assessed by manually reading the event descriptions, which are herein all termed as "PUT". A PUT was classified as either suicide or accident, as judged by investigative operatives at SL, whom analyzed the behavioral intent of each PUT event captured on CCTV video recordings (available at all stations since 2010). This information was then complemented a systematic classification procedure which also involved other entities not related to the metro operator, such as forensic/accident investigators and the police [4]. For the purposes of this study, the primary outcome comprised only PUT events determined to be due to suicidal intent, excluding accidental PUT events. We included only PUT which occurred through access from station platforms. PUT among rail workers was excluded. Secondary outcomes were: (1) death by suicide due to PUT, which amounted to about half of all primary outcome PUT and was here specified as a key secondary outcome; (2) individuals safeguarded for suicide risk; (3) cancelled train-traffic kilometers due to trespassing and PUT, as well as (4) PUT due to accidents. Further details are found in Additional File 1.

### Statistical analysis

Following recommended practice [12], we pre-specified a controlled interrupted time series (CITS) analysis, which consists of an interrupted time series (ITS) segmented regression analysis of intervention stations, adjusted for the concurrent level-changes at untreated control stations. The CITS approach was selected as it helps to account for other time-varying confounders in the metro system [11–13]. The intervention-related change was assumed to result in an immediate reduction (level change) in the number of PUT due to suicidality, rather than a gradual change over time (trend change). As this study used a natural experiment design, external factors mainly determined the number of time points. Quarterly data were used to increase events per time point, to obtain more reliable model estimates. The pre-intervention period included 47 time points, followed by 4 time points in the post-intervention period with AI-CCTV exposure (Table 1). An extended post-period contained an additional 11 time points (Table 1), but had several other known concurrent external events, e.g. perpetual media exposure about the AI-CCTV intervention. Analyses were conducted on complete data with no missing observations. All 86 stations in the Stockholm metro without AI-CCTV constituted the control group, which was exposed to similar concurrent events as the intervention stations, i.e. same exposure to system-wide and societal factors, e.g. the COVID-19 pandemic, population dynamics, and health care conditions in Stockholm [11]. Indeed, both intervention and control stations fulfilled the common trend requirement of CITS in the pre-intervention time period [11]. The primary PUT outcome CITS and ITS results were followed up post hoc, using a series of pre-specified sensitivity analyses with alternative model specifications and testing secondary outcomes (Table 1). The alternative model specifications were without adjustment for underlying time trends, i.e. controlled difference-in-differences (DID) and uncontrolled pre-post comparisons, which are commonly used to evaluate population-level interventions for suicide prevention [15]. General trends were visualized using locally weighted scatter plot smoothing (LOESS), which is a recommended non-parametric regression method [11]. Further pre-specified post hoc analyses assessed potential displacement from intervention stations to nearby control stations. Robustness to unmeasured confounding was evaluated using E-values, calculated with an online E-value calculator [16, 17]. All segmented regression models were performed in Stata SE versions 17.0 and 18.5, using Poisson for count outcomes, robust standard errors and a one-tailed directional hypothesis, as we expected a reduction. There was no evidence found of excess autocorrelation or non-stationarity in either the time series data or GLM model residuals. More details are found in Additional Files 1 and 2.

We repeated the primary and key secondary outcome analyses with Bayesian inference using weakly informative priors, to mitigate putative sparse-data problems with the models and to gain verification of the direction and magnitude of the effect [18]. A recent meta-analysis on the effects of means restriction against suicide deaths yielded *IRR* = 0.09 (95% CI 0.04–0.21) [19]. We here tested *IRR* 95% normal ranges of 0.1–10, 0.05–20, 0.01–100, as well as the default, very weak prior in Stata (100 standard deviations, corresponding to 10^–85^—10^85^ 95% *IRR* interval). The prior mean was *IRR* = 1 for no effect. The posterior probability (*IRR* < 1 | data) results reported are naturally one-tailed and the probability that there was an effect is here also referred to as: < 0.8, insufficient to low probability; 0.8–0.9, moderate probability; 0.9–0.95, moderately high probability; > 0.95, high probability. More details are found in Additional Files 1 and 2.

## Results

### Reductions in PUT due to suicidality following AI-CCTV implementation

**Fig. 2:**
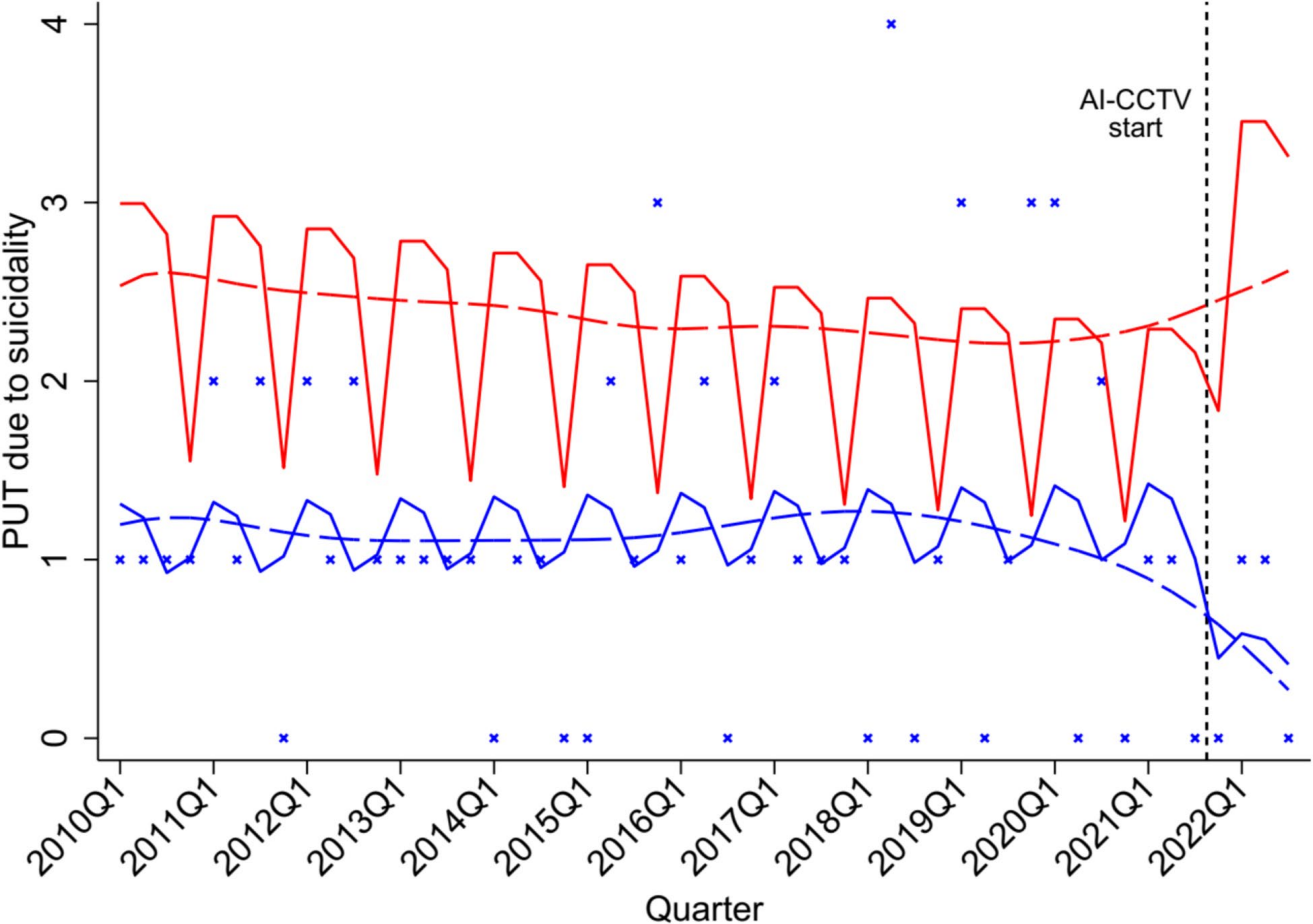
Pre-post time series ITS models. The ITS predicted mean curves of quarterly number of PUTs due to suicidality in intervention (blue line) and control (red line) stations. Dashed lines depict the LOESS trend curves of the quarterly PUT outcome counts at AI-CCTV intervention (blue x-marks) or control stations (not depicted). The vertical dashed line depicts the implementation onset of the AI-CCTV intervention at the Q3/Q4 2022 boundary. In the primarily tested CITS level-change, the red line acts as the counterfactual for the blue line

Before the AI-CCTV implementation, the average number of PUT due to suicidality was 1.17 per quarter in the intervention group (55 in total) and 2.30 per quarter in the control group (108 in total). After the introduction of AI-CCTV, these numbers changed to 0.50 in the intervention group (2 in total) and 3.00 in the control group (12 in total), as displayed in Fig. 2. Event counts were low, particularly in the post-intervention period, and quarter-level averages are therefore sensitive to a small number of events.

In the primary CITS analysis, AI-CCTV implementation was associated with a lower rate of person-under-train (PUT) events related to suicidality (*IRR* = 0.32, 90% CI 0.012 to 0.80; one-sided *p* = 0.021), adjusted for time-varying confounders and seasonality. According to E-value, this association was moderately robust to any equally timed confounder affecting outcome and exposure (Additional File 1). An uncontrolled ITS model restricted to the intervention stations alone, also showed a significant reduction (*IRR* 0.41, 90% CI 0.17 to 0.96; one-sided *p* = 0.043; Fig. 2). Subsequent sensitivity analyses supported the observation of lower rates following implementation (Table S5 in Additional File 1). In contrast, ITS of the control group alone showed a non-significant increase after AI-CCTV implementation (*IRR* = 1.54; 90% CI 0.87–2.74; *p* = 0.11; Fig. 2), thus the inversed effect contributed to the enhanced CITS point estimate of 0.32 as compared to the ITS estimate of *IRR* = 0.41. However, we observed no significant displacement to nearby control stations in the post period, as PUT counts at stations adjacent to those with AI-CCTV remained similar to those at more distant control stations (Tables S5 and S6 in Additional File 1). During the post-intervention period, one PUT event was not detected due to improvable CCTV limitations (insufficient image resolution); excluding this event resulted in a stronger, short-term level change (CITS *IRR* = 0.16; Tables S5 in Additional File 1).

### Secondary outcomes

We next tested secondary outcomes using only simplified pre-post and benchmark DID models, to mainly examine consistency in the direction of the estimated change (Additional File 1). Death by suicide following PUT due to suicidality was a key secondary outcome. Before implementation, suicides occurred at both intervention (n = 28 suicides out of n = 55 PUT) and control stations (n = 69 suicides out of n = 108 PUT). During the post-implementation period, no suicides occurred at stations with AI-CCTV (n = 0 suicides out of n = 2 PUT), whereas six suicides were observed at control stations (n = 6 suicides out of n = 12 PUT), resulting in point estimates of *IRR* = 0.3 (*p* < 0.25) (see Table S7 in Additional File 1). The number of individuals safeguarded due to suicidality was examined as an intermediate outcome reflecting overall safeguarding activity, showed point estimates of *IRR* = 0.8. PUT due to other reasons than suicidality, e.g. accidents, showed point estimates of *IRR* = 0.2—0.5. Finally, the number of cancelled train-kilometres in the metro system overall were significantly lower after AI-CCTV implementation.

We also list all AI-CCTV triggered alarms that involved a person being safeguarded and resulted in operational responses such as train speed reductions, train stops, or power disconnections. By Q2 2025, SL had recorded 36 such events, which may or may not have resulted in PUT in the absence of the AI-CCTV alarm. In addition, all cases of PUT due to suicidality which nevertheless occurred at stations with AI-CCTV are also described, involving mainly platform jumps immediately before train arrival or technical limitations affecting detection (Additional File 1).

### Bayesian sensitivity analysis

Putative problems may arise because of sparse data, i.e. due to the relatively low counts in the time series, the short primary post-period and then particularly if using CITS and ITS models with multiple parameters. We therefore complemented the primary PUT outcome analyses by Bayesian estimation using weak priors, providing estimates which are robust to sparse data biases (Table 2). Among the models that used the control group (CITS and DID), the *IRRs* were in the range of 0.27–0.43 and the posterior probabilities were moderately high to high (Table 2). Among the models that did not use the control group (ITS and Pre/post), the *IRRs* were in the range of 0.32–0.49 and the posterior probabilities were moderately high (Table 2). In addition, we also performed a Bayesian estimation for the even more sparse key secondary outcome, i.e. death by suicide, which showed *IRR* ~ 0.2 for both the controlled (DID) and uncontrolled (Pre/post) estimates, as well as posterior probabilities which were moderately high to high (Table S7 in Additional File 1). The small changes to the *IRR* estimates using different weak prior choices compared to the ordinary Poisson regression (Table 2), did not suggest the existence any severe sparse data biases for the models used here. Results instead provided additional evidence that a reduction in both PUT and also death by suicide outcomes were moderately to highly probable, with an effect magnitude of *IRR* ≤ 0.5.

**Table 2:** Bayesian analysis using weakly informative priors to mitigate putative sparse-data problems

Model	Observed *IRR*^a^	Prior, 95% *IRR* ranges		Bayesian results			
			Maximum Gelman-Rubin quality metric	ESS	Posterior median *IRR*^*b*^	% change in IRR estimate^*c*^	Posterior probability (*IRR* < 1 | data)
CITS	0.3120	10^–85^—10^85^	1.027^d^	573^d^	0.2429	−22.1%	0.970
CITS	0.3120	0.01–100	1.005	4432	0.3082	−1.2%	0.961
CITS	0.3120	0.05–20	1.003	4629	0.3549	13.8%	0.955
CITS	0.3120	0.1–10	1.004	4359	0.4061	30.2%	0.938
ITS	0.4084	10^–85^—10^85^	1.086^d^	288^d^	0.3179	−22.2%	0.930
ITS	0.4084	0.01–100	1.005	4406	0.3816	−6.6%	0.922
ITS	0.4084	0.05–20	1.003	5168	0.4403	7.8%	0.908
ITS	0.4084	0.1–10	1.004	5468	0.4834	18.4%	0.895
DID	0.3273	10^–85^—10^85^	1.002	2688	0.2717	−17.0%	0.960
DID	0.3273	0.01–100	1.003	4862	0.3227	−0.5%	0.953
DID	0.3273	0.05–20	1.001	5053	0.3798	5.2%	0.941
DID	0.3273	0.1–10	1.001	4576	0.4261	9.9%	0.926
Pre/post	0.4273	10^–85^—10^85^	1.002	3903	0.3547	−17.0%	0.938
Pre/post	0.4273	0.01–100	1.001	7367	0.3894	−3.8%	0.931
Pre/post	0.4273	0.05–20	1.000	7636	0.4415	1.4%	0.920
Pre/post	0.4273	0.1–10	1.000	7954	0.4853	5.8%	0.910

*CITS* Controlled interrupted time series (with time trend and controls), *DID* Difference-in-differences (without time trend and with controls), *ESS* Effective sample size, *IRR* Incidence rate ratio, *ITS* Interrupted time series (with time trend and without controls), *MCMC* Markov Chain Monte Carlo, Pre/post, after vs before ratio (without time trend or controls)

^a^*IRR* for the intervention from ordinary Poisson regression

^b^Bayesian median estimate of *IRR* in the posterior distribution across all four chains each having *N* = 10,000 MCMC sample size

^c^Difference in Bayesian median estimate of *IRR vs IRR* from ordinary Poisson regression

^d^insufficient MCMC sampling quality

### Exploratory extended post-period analysis

Results from an exploratory extended post-period analysis are presented in Additional File 1. Because this period included multiple heterogeneous system-wide exposures, including media reporting and additional safety interventions, these analyses were interpreted separately from the primary analysis and were not used to assess the specific contribution of AI-CCTV. Reduced rates of PUT events due to suicidality at AI-CCTV stations was still observed in the primary post-period per se, although for the combined extended post-period, the CITS level-change estimate was no longer statistically significant. From Q4 2022 onward, a decreasing trend was instead observed across the entire metro system, suggesting that changes during the extended period may reflect broader system-wide influences, rather than effects by AI-CCTV alone.

## Discussion

This study provides preliminary findings of a preventive effect on suicide and suicide attempts from the implementation of AI-CCTV in the Stockholm metro. However, the short post-intervention period, low event counts, and a non-significant post-intervention increase in the control group warrant cautious interpretation. We used a controlled interrupted time-series design to account for system-wide changes over time and secondary outcome measures that yielded directionally consistent estimates, supporting a preventive effect, but they do not provide definitive causal evidence or information about long-term effectiveness. We also reported uncontrolled ITS and pre-post results alongside the controlled analysis, as part of a recommended robustness check in methodological guides [12]. The post-intervention increase in the control group increased the relative effect estimates when using the CITS-model. We therefore interpret the controlled estimate cautiously and alongside the absolute reduction observed at the intervention stations and the uncontrolled ITS estimate for intervention stations. Finally, a Bayesian sensitivity analysis excluded the possibility of major sparse data biases contributing to the results reported here.

Use of a controlled design was particularly important given major time-varying influences during the study period, including the COVID-19 pandemic, which affected travel patterns and train service operations. In the CITS, the control group provided the broadest available system-level counterfactual. Although PUT incidents due to suicidality increased in the control group during the post-period, this increase was not statistically significant. Spillover to control stations is an important concern when interpreting the controlled estimate. We therefore examined the most plausible local spillover pattern in a sensitivity analysis. This analysis was intended to assess local spillover to adjacent stations, but it cannot exclude more complex or broader network-level displacement. Control stations one stop from an AI-CCTV station did not show a larger pre-to-post increase than stations two or more stops away. The intervention was not announced to passengers at the stations. The only publicly available information about which stations had AI-CCTV was on SL’s website, which would likely require active searching and prior planning for an individual considering a suicide attempt. Taken together, these findings do not support local leakage from intervention stations to nearby control stations. However, more complex or broader network-level displacement cannot be ruled out, and the estimates should therefore be interpreted alongside their uncertainty, the absolute changes at intervention stations, and the consistency across controlled and the uncontrolled ITS estimate. Displacement is not only a concern when interpreting potential spillover to a control group, but it's also fundamentally related to the relevance of using means restriction in public places. A recent meta-analysis assessing this did not find any clear evidence that means restriction at high-risk locations generally leads to increases in suicides at other sites [19]. However, even if AI-CCTV acted by causing displacement to other stations in the metro stations, the reduction thus still existed at the intervention stations per se and it could also suggest the importance of further use of AI-CCTV in the entire metro system.

Placebo analyses with a false intervention start showed no evidence of a comparable change before implementation, supporting the interpretation that the observed reduction was temporally aligned with the AI-CCTV implementation. E-values suggested that strong unmeasured confounding would be required to move the point estimate to the null, although more modest confounding could shift the confidence interval to include the null [16]. The number of events was sparse, particularly after implementation, so quarterly averages and the apparent post-period increase in the control series are sensitive to a small number of incidents and should not be overinterpreted as a systematic change in risk.

Death by suicide, here pre-specified as a key secondary outcome, also showed a reduction consistent with the primary findings on the broader PUT due to suicidality. Noteworthy, no suicides occurred at stations equipped with AI-CCTV during the primary post-implementation period of one year, in contrast to six suicides at the control stations. Given the small numbers and the fact that lethality in PUT incidents depends on situational factors beyond intent, these results should be interpreted cautiously. Indeed, about half of PUT due to suicidality did not result in deaths, i.e. could thus be classified as suicide attempts. Nonetheless, a reduction in PUT incidents could plausibly be followed by fewer fatal outcomes as a downstream consequence. In this study the direction and magnitude of the change in suicide deaths were consistent with the primary result.

The number of individuals safeguarded due to suicidality did not show a clear intervention-associated change relative to control stations. This outcome reflects overall safeguarding activity and does not specifically capture the most acute high-risk situations, i.e. standing at the platform edge or trespassing. Descriptive analyses of high-risk situations illustrated that AI-CCTV enabled operational responses, including train speed reduction and train stopping. These findings provide insight into system functioning and potential mechanisms, rather than evidence of a change in the frequency of safeguarding.

PUT due to accidents also showed a change in the same direction following implementation, although uncertainty was even more substantial due to even fever event counts. This pattern is plausible given that AI-CCTV targets behaviours and situations at the platform edge that are common precursors not only to suicidal acts, but also to accidental falls and track intrusions. Train cancellations related to person-under-train events and trespassing were lower following implementation of AI-CCTV, with consistent reductions observed in both pre–post and DID analyses. These system-level changes are relevant from operational and public health perspectives, given the substantial societal costs associated with major service disruptions in rail-bound traffic.

Previous research has shown that a small set of basic behaviours, such as entering the track area and remaining very close to the platform edge, frequently precede PUT, and these findings directly informed the design of the AI-CCTV system evaluated here [20]. Evidence from other settings indicates that behavioural patterns preceding suicide attempts can vary across systems and cultural contexts. In the Montreal metro, behaviours such as pacing between the platform edge and the wall and leaving objects behind were associated with elevated suicide risk [21]. However, such behaviours were uncommon in the Stockholm setting [20], which highlight the importance of training the AI models on locally prevalent behaviours clearly increase proximal PUT risks, such as entering the track area or remaining at the platform edge.

Several PUT due to suicidality which occurred despite the AI-CCTV usage, were related to very late platform jumps immediately before train arrival or to technical limitations, such as incomplete camera coverage or insufficient resolution. Sensitivity analyses excluding events affected by camera limitations yielded stronger preventive estimates, suggesting that system performance could potentially be improved, e.g., through technical upgrades of the cameras’ surveillance area over the platforms and using a more automated process, e.g., by visual signals on the track or alerts directly into to the drivers cabin, to alert drivers if an elevated risk is detected without having to be manually assessed by a safety officer. Publicly available alarm buttons in the platform area, as used, e.g., in the Tokyo metro [22], are another potential enhancement to prevent jumps that occur immediately before train arrival. This is as a straightforward complementary method enabling faster stopping of trains approaching the station when a PUT risk is detected by bystanders.

AI-CCTV shares features with other public health interventions based on surveillance and early detection [10]. It is also functions as a partial means-restriction measure, comparable to platform-end lengthwise fencing [23] and mid-track fencing [24]. Unlike static physical barriers, AI-CCTV can be viewed as a dynamic means of restriction, activating protective responses only when an elevated risk is detected. In this sense, AI-CCTV operates in the late pre-crash or early crash phases described by Haddon [25]. AI-CCTV also extends beyond means restriction, since the detection aspect enable subsequent human interventions for the individuals at risk who trigger the system, thereby increasing the likelihood that they are referred to healthcare for appropriate treatment. The preliminary results from this study may be generalizable to station settings similar to the one described in the Additional File 1. The time interval between a system alert and the ability to intervene on the detected suicidal behavior is crucial, as was also discussed in a recent systematic review of surveillance system [10]. For example, a video-based alert system of cars that stop on a bridge in South Korea, did not manage to reduce deaths by suicide, whereas improved physical barrier on the bridge fence did reduce deaths by suicide [26]. Intervening on detected suicide behavior on a bridge may be more difficult to do fast enough, compared to the rail settings where one can instantly remove the means by simply stopping the train. From a broader suicide prevention perspective, interventions in transport environments should complement, not replace, indicated preventive efforts such as access to healthcare and psychosocial support.

Strengths of this study include the use of a CITS time series analysis aiming to control for external time-varying confounders. The AI-CCTV intervention was clearly defined spatiotemporally and was also described in detail, including its technical configuration, detection logic, and operational workflow, enabling reproducibility in practice. Multiple pre-specified sensitivity analyses and related secondary outcomes showed findings consistent with the primary results. The long pre-intervention period strengthened the assessment of baseline secular time trends and analyzing all PUT events in the Stockholm metro lowered the risk of selection bias.

A key limitation was that the post-period data only covered four quarters, which reduced precision, although this is more of a problem for trend, rather than level change estimates [27]. Another limitation was an unexplained, non-significant increase in events in the control group in the post-period, which may have artificially lowered the risk ratio and exaggerated the intervention's benefits. The non-significant increase in the control group should not be interpreted as clear evidence that the underlying risk of PUT due to suicidality increased at control stations, although broader network-level displacement or other time-varying differences between intervention and control stations cannot be ruled out. This increase may have inflated the relative CITS point estimate. We therefore interpret the estimated level change from the CITS model cautiously. However, the absolute reduction at intervention stations and the uncontrolled ITS estimate for intervention stations both support a possible preventive effect of AI-CCTV. The uncontrolled ITS estimate is not affected by the post-intervention increase in the control group, but do not control for system-wide time-varying confounding.

We assessed the level change at implementation and then validated the direction of the change using less data-intensive, pre-post sensitivity analyses, which do not rely on accurate trend estimates. Nevertheless, a longer follow-up period without heterogeneous exposures would, in principle, have been preferred to obtain more information about the durability of the level change. Another limitation was the quarterly aggregation used to reduce noise due to the outcome being rare, at the cost of temporal resolution. Despite this, the event counts were still sparse in the time series. This limitation was also partly addressed by primarily testing for level changes rather than trend changes, and by adding a Bayesian sensitivity analysis to assess whether the estimated change was robust to sparse data biases.

These findings indicate that AI-CCTV should be treated as an operational intervention rather than simply a detection tool. Its effect will probably depend on how well detection is linked to operational responses that rapidly mitigate the risk. This includes camera coverage of platform areas, especially near the track, the time from elevated-risk detection to action and whether train braking can be initiated quickly when an acute risk is detected. In situations where the danger is immediate, for example, when a person is on the track, automatic signaling directly to the train driver could shorten the response time. At the same time, parallel manual review may still have value, particularly for verifying alarms, contacting emergency services, and ruling out false positives, although it may not be required for all high-risk alerts if the system is well-functioning and has few false positives. AI-based CCTV may be especially useful on open platforms where physical barriers are difficult, costly, or not currently planned, but it should be seen as a complement to other suicide prevention measures in rail environments, not as a replacement or a standalone intervention.

Future implementation should be planned to enable evaluation. In the present study, the post-intervention period was short because the interventions were communicated intensively in mass media one year after their implementation. Such attention makes robust evaluation of effects more difficult. Future rollouts should therefore avoid public communication of perceived early success, to instead extend the post-periods for more robust evaluations. Future studies should examine whether the short-term effects observed in this study are sustained over longer follow-up in other systems. Future studies should quantify the relative contribution of detecting persons entering the track area and persons standing immediately at the platform edge to preventing PUT events. Furthermore, one should study whether similar effects are found when AI-CCTV is introduced across an entire rail system and how the intervention performs in rail systems with higher train speeds, where the time available for intervention is shorter. Studying whether more complex functionality in an AI-CCTV system, such as assessing emotions from facial expressions, could benefit suicide prevention in rail systems, would also be of interest. It would also be valuable to examine whether similar preventive effects can be achieved with other sensor types, such as LiDAR, rather than only using camera-based surveillance. One potential risk and drawback of this kind of intervention that future studies should address is whether system-wide implementation changes the characteristics of suicidal events, including the timing before a jump from a platform in relation to train arrival, train speed, and the practical window for intervention. Ethical issues and public acceptability also need closer attention, including how passengers and staff perceive AI-based surveillance for suicide prevention in public transport.

## Conclusion

In the study reported here, implementation of AI-CCTV suggested a preventive effect on PUT due to suicidality at Swedish metro stations, i.e. suicide attempts and deaths by suicide. To our knowledge, this study represents the first empirical evaluation of an AI-based suicide prevention intervention in a rail-based transport system. More broadly, this study contributes to an emerging field of AI-based surveillance interventions for suicide prevention, where empirical evidence remains limited. From a public health perspective, AI-enabled real-time detection could contribute to a population-level risk reduction in rail and perhaps other environments, when timely operational responses or restriction of means in high-risk situations are possible. Given its scalability and potential for broader implementation as compared to full physical barriers, AI-CCTV may have potential to become a broader intervention for suicide prevention in rail-bound traffic worldwide.

## Supplementary Information

Additional file 1. Supplementary appendix.

Additional file 2. Analyzed data and Stata commands.

## Data Availability

Refer to Additional file 2.

## Abbreviations

AI-CCTV: Artificial Intelligence based Closed-Circuit TeleVision
CITS: Controlled Interrupted Times Series
DID: Difference-In-Differences
ESS: Effective sample size
IRR: Incidence Rate Ratio
ITS: Interrupted Times Series
LOESS: LOcally weighted Scatter plot Smoothing
MCMC: Markov Chain Monte Carlo
PUT: Persons Under Train
Q: Calendar quarter
SL: Storstockholms Lokaltrafik

## References

[1] World Health Organization. Suicide worldwide in 2021: global health estimates. 2025.

[2] Davis Weaver N, Bertolacci GJ, Rosenblad E, Ghoba S, Cunningham M, Ikuta KS, et al. Global, regional, and national burden of suicide, 1990–2021: a systematic analysis for the Global Burden of Disease Study 2021. Lancet Public Health. 2025;10:e189-202. 10.1016/S2468-2667(25)00006-4. 39986290 10.1016/S2468-2667(25)00006-4 PMC11876099

[3] Eurostat. Railway safety statistics in the EU. 2024. https://ec.europa.eu/eurostat/statistics-explained/index.php?title=Railway_safety_statistics_in_the_EU. Accessed 11 Dec 2025.

[4] Andersson A-L, Liss G, Sokolowski M. Accident or suicide? New registration procedures and improved classification of suicide vs accident deaths on the Swedish railways: an interrupted time series analysis of years 2000–2023. Transport Res Interdiscip Perspect. 2025;34:101650. 10.1016/j.trip.2025.101650.

[5] Fredin-Knutzén J, Olsson N, Rosberg T, Thorslund B, Lidestam B. Train drivers’ work related stress and job satisfaction. J Occup Environ Med. 2023;65:775–82. 10.1097/JOM.0000000000002903. 37311076 10.1097/JOM.0000000000002903

[6] Belur P, Sherry P, Rodriguez I, Kurkure C, Joshi SV. Strategies for reducing suicide at railroads: a scoping review of evidence and gaps. IJERPH. 2024;22:18. 10.3390/ijerph22010018. 39857471 10.3390/ijerph22010018 PMC11765353

[7] Zalsman G, Hawton K, Wasserman D, Van Heeringen K, Arensman E, Sarchiapone M, et al. Suicide prevention strategies revisited: 10-year systematic review. Lancet Psychiatry. 2016;3:646–59. 10.1016/S2215-0366(16)30030-X. 27289303 10.1016/S2215-0366(16)30030-X

[8] Clarke RV, Poyner B. Preventing suicide on the London underground. Soc Sci Med. 1994;38:443–6. 10.1016/0277-9536(94)90445-6. 8153749 10.1016/0277-9536(94)90445-6

[9] International Union of Railways. UIC Opt-In Project “AI4SAFEBEHAVE” kick-off meeting held. UIC Communications. 2024.

[10] Joyner L, Cliffe B, Mackenzie J-M, Hawton K, Craig P, Marzano L. The effectiveness of surveillance technology for the prevention of suicides in public spaces: a systematic review. medRxiv. 2025;:2025.10.17.25338217. 10.1101/2025.10.17.25338217. 10.1136/bmjph-2025-004286 PMC13330960 42403671

[11] Bottomley C, Scott JAG, Isham V. Analysing interrupted time series with a control. Epidemiologic Methods. 2019;8:20180010. 10.1515/em-2018-0010.

[12] Lopez Bernal J, Cummins S, Gasparrini A. The use of controls in interrupted time series studies of public health interventions. Int J Epidemiol. 2018;47:2082–93. 10.1093/ije/dyy135. 29982445 10.1093/ije/dyy135

[13] Linden A. Conducting interrupted time-series analysis for single- and multiple-group comparisons. Stata J. 2015;15:480–500. 10.1177/1536867X1501500208.

[14] Von Elm E, Altman DG, Egger M, Pocock SJ, Gøtzsche PC, Vandenbroucke JP. The Strengthening the Reporting of Observational Studies in Epidemiology (STROBE) Statement: Guidelines for Reporting Observational Studies. Epidemiology. 2007;18:800–4. 10.1097/EDE.0b013e3181577654. 18049194 10.1097/EDE.0b013e3181577654

[15] Spittal MJ, Gunnell D, Sinyor M, Clapperton A, Roberts L, Pirkis J, et al. Evaluating population-level interventions and exposures for suicide prevention. Crisis. 2024. 10.1027/0227-5910/a000961. 38770800 10.1027/0227-5910/a000961 PMC11783171

[16] VanderWeele TJ, Ding P. Sensitivity analysis in observational research: introducing the E-value. Ann Intern Med. 2017;167:268–74. 10.7326/M16-2607. 28693043 10.7326/M16-2607

[17] Mathur MB, Ding P, Riddell CA, VanderWeele TJ. Web site and r package for computing E-values. Epidemiology. 2018;29:e45–7. 10.1097/EDE.0000000000000864. 29912013 10.1097/EDE.0000000000000864 PMC6066405

[18] Hamra GB, MacLehose RF, Cole SR. Sensitivity analyses for sparse-data problems-using weakly informative bayesian priors. Epidemiology. 2013;24:233–9. 10.1097/EDE.0b013e318280db1d. 23337241 10.1097/EDE.0b013e318280db1d PMC3607322

[19] Too LS, Shin S, Taouk Y, Pirkis J, Sinyor M, Yip PSF, et al. Impact of interventions at frequently used suicide locations on occurrence of suicides at other sites: a systematic review and meta-analysis. Psychol Med. 2025;55:e168. 10.1017/S0033291725100792. 40551600 10.1017/S0033291725100792 PMC12201958

[20] Ceccato V, Wiebe DJ, Vrotsou K, Nyberg U, Grundberg A. The situational conditions of suicide in transit environments: an analysis using CCTV footage. J Transp Health. 2021;20:100976. 10.1016/j.jth.2020.100976.

[21] Mishara BL, Bardon C, Dupont S. Can CCTV identify people in public transit stations who are at risk of attempting suicide? An analysis of CCTV video recordings of attempters and a comparative investigation. BMC Public Health. 2016;16:1245. 10.1186/s12889-016-3888-x. 27974046 10.1186/s12889-016-3888-x PMC5157080

[22] Tokyo Metro. What to do if... https://www.tokyometro.jp/lang_en/corporate/safety/safety_pocketguide/emergency/index.html. Accessed 11 Mar 2026.

[23] Fredin-Knutzén J, Hadlaczky G, Wigren A, Sokolowski M. A pilot study evaluating the preventive effects of platform-end lengthwise fencing on trespassing, person struck by train and traffic delays. J Safety Res. 2024;88:78–84. 10.1016/j.jsr.2023.10.010. 38485387 10.1016/j.jsr.2023.10.010

[24] Fredin-Knutzén J, Hadlaczky G, Andersson A-L, Sokolowski M. A pilot study evaluating the effectiveness of preventing railway suicides by mid-track fencing, which restrict easy access to high-speed train tracks. J Safety Res. 2022;83:232–7. 10.1016/j.jsr.2022.08.019. 36481013 10.1016/j.jsr.2022.08.019

[25] Haddon W. A logical framework for categorizing highway safety phenomena and activity. J Trauma Inj Infect Crit Care. 1972;12:193–207. 10.1097/00005373-197203000-00002 5012817

[26] Shin S, Pirkis J, Spittal MJ, Too LS, Clapperton A. Change in incidents of suicidal acts after intervention on a bridge in South Korea. Soc Psychiatry Psychiatr Epidemiol. 2025;60:685–91. 10.1007/s00127-024-02744-9. 39120715 10.1007/s00127-024-02744-9 PMC11870871

[27] Baicker K, Svoronos T. Testing the Validity of the Single Interrupted Time Series Design. Cambridge, MA: National Bureau of Economic Research; 2019. 10.3386/w26080.

