# Supplementary material for: Reduction in suicides and suicide attempts following implementation of AI-based video surveillance in the Stockholm metro system: an intervention study
Source: BMC Public Health. 2026 Jul 1;26:1997. doi: 10.1186/s12889-026-28358-1 (PMC13321902; doi:10.1186/s12889-026-28358-1)
Supplement: Supplementary file 1 — Additional file 1. Supplementary appendix. [file 12889_2026_28358_MOESM1_ESM.docx]

Supplementary Appendix

**Reduction in suicides and suicide attempts following implementation of AI-based video surveillance in the Stockholm metro system: an intervention study**

Table of Contents

[Supplementary introduction 2](#_Toc231062598)

[AI-CCTV 2](#_Toc231062599)

[Information for travellers about the system 4](#_Toc231062600)

[SL’s other measures for suicide prevention in the metro 5](#_Toc231062601)

[Helpline signage 5](#_Toc231062602)

[Enhanced Security Information 6](#_Toc231062603)

[Blue LED-lights 7](#_Toc231062604)

[Map of interventions 9](#_Toc231062605)

[The Stockholm Metro system characteristics 9](#_Toc231062606)

[AI-CCTV media attention 11](#_Toc231062607)

[Supplementary methods 14](#_Toc231062608)

[STROBE statement 14](#_Toc231062609)

[Classification of PUT incidents 18](#_Toc231062610)

[Statistical analysis 18](#_Toc231062611)

[Pre-intervention diagnostics 18](#_Toc231062612)

[Dispersion 19](#_Toc231062613)

[Autocorrelation and stationarity 19](#_Toc231062614)

[Seasonal trends 19](#_Toc231062615)

[E value 19](#_Toc231062616)

[Sensitivity analyses 20](#_Toc231062617)

[The extended post-intervention period 21](#_Toc231062618)

[Statistical analysis of secondary outcomes 22](#_Toc231062619)

[Supplementary results 24](#_Toc231062620)

[Primary outcome: additional analyses 24](#_Toc231062621)

[Secondary outcomes 26](#_Toc231062622)

[Time periods for secondary outcome 26](#_Toc231062623)

[Secondary outcome analysis 26](#_Toc231062624)

[Exploratory analysis of the extended post-period 31](#_Toc231062625)

[References 35](#_Toc231062626)

# Supplementary introduction

## AI-CCTV

Information in this section is based on personal communication with Mikaela Pettersson and Steffen Muschter at SL (January 2026), except where a published source is referenced. The AI-CCTV was installed on eight underground stations and six outdoor stations in the Stockholm metro (Fig. S8); an additional underground station was equipped during the extended post-period (after i.e. Q3 2022). The software used was IRIS-SL, a video analytics solution developed by Irisity [1], which analyzes video streams from existing CCTV cameras installed at the stations prior to the intervention.

The AI-CCTV system was purchased through a procurement process. As part of the procurement, SL provided tenderers with descriptions of relevant scenarios and specifically developed test material. The test material included video footage of persons walking along the track area while dressed in warning clothing, as well as human-sized dolls that were thrown onto the tracks to simulate fall accidents. Around 70 examples were provided for each scenario. Apart from this, the procurement intentionally did not specify any particular type or amount of training data. This was done to avoid excluding newer detection methods that may not have been known to the client at the time of procurement.

The AI component is a trained model that interprets the video feed and integrates signals over time and context, rather than relying solely on fixed thresholds. It handles real-world variability by detecting and tracking people regardless of lightning and weather conditions. Based on these detections, movement patterns are analyzed, and if given thresholds are exceeded, alarms are generated. This, together with an AI model for object detection, reduces false alarms and helps capture ambiguous, sequential risk patterns that rule-based criteria struggle to handle.

Initially, the AI-CCTV system included features that detected when a person (1) entered the track area (trespassing) or stood immediately at the platform edge, the latter of which is the system feature that was evaluated in this article. In May 2023 (during the extended post-period), additional features were introduced, enabling detection of persons who (2) stood anywhere within the marked safety zone, painted white on the platform, for more than 20 seconds, (3) remained seated on a bench for more than 20 minutes, or (4) were lying down anywhere on the platform (Fig. S1). The camera’s field of view covered the whole platform and 4-6 cameras were required for each platform. The cameras were located in the inner ceiling of the stations ≈ 3-5 meters above the floor, and were always located above the platform near the track area. Figures S1 and S2 illustrate the alarm logic of the AI-CCTV system, including area definitions and time-based trigger conditions used across stations.

The software cost over a five-year period, had a maximum of 4 million SEK (approximately 370,000 EUR) [1], corresponding to approximately 74,000 EUR per year. In addition, the AI-CCTV system required an additional staff of approximately 25% of a full-time duty, monitoring incoming alarms for 21.5 hours per day on weekdays and 24 hours on weekends. The annual cost for staff amounts to approximately 50,000 EUR.


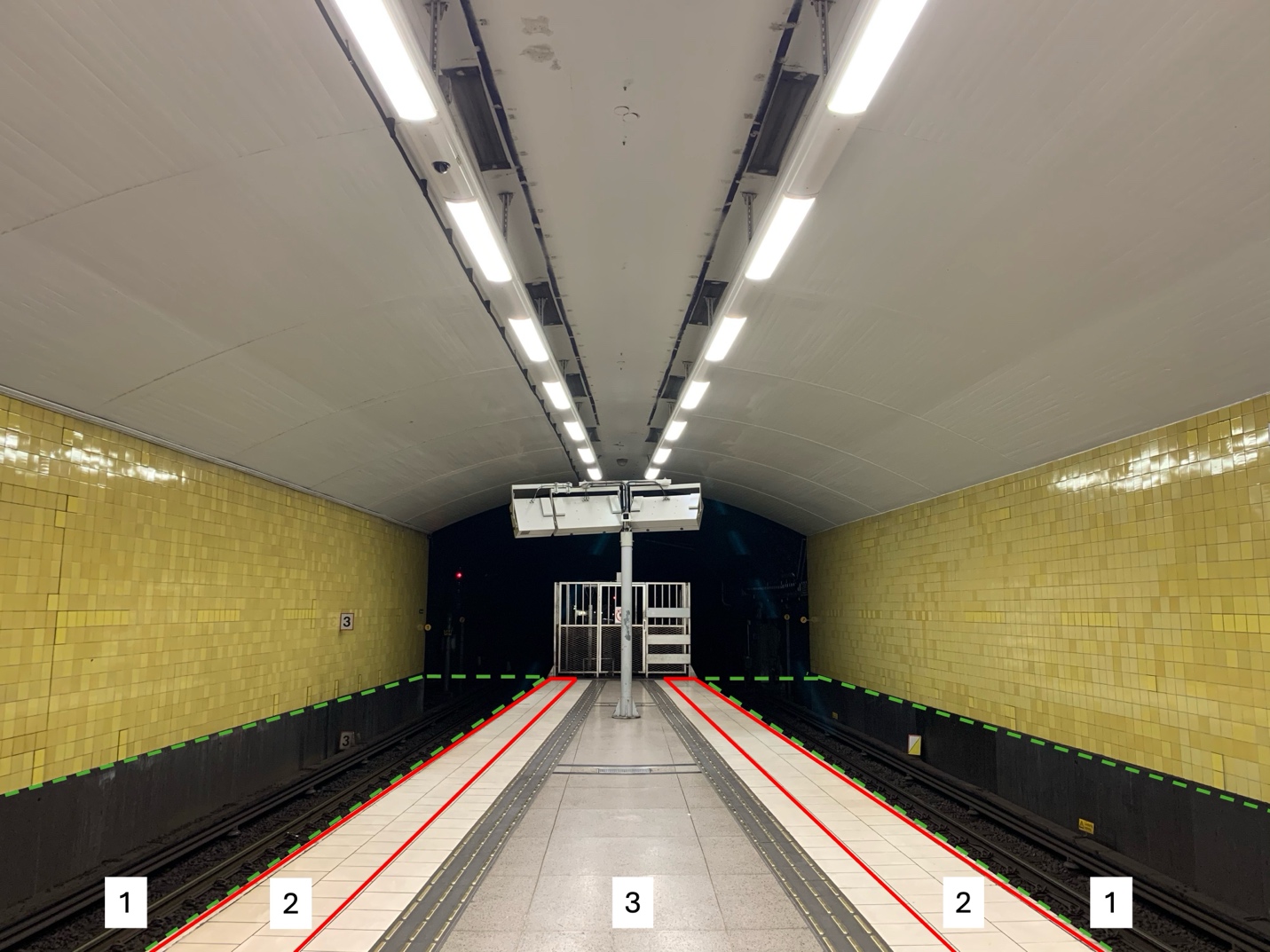


**Fig. S1.** Principle illustration of how the alarm zones were defined in the system. The first version of the AI-CCTV system triggered an alarm 5 seconds after at least 70 % of an individual’s body entered Area 1. In a later system update, additional alarm criteria were introduced, if the middle and one corner of the person’s base outline remained within Area 2 for more than 20 seconds, or sat on a bench located within Area 3 for more than 20 minutes.


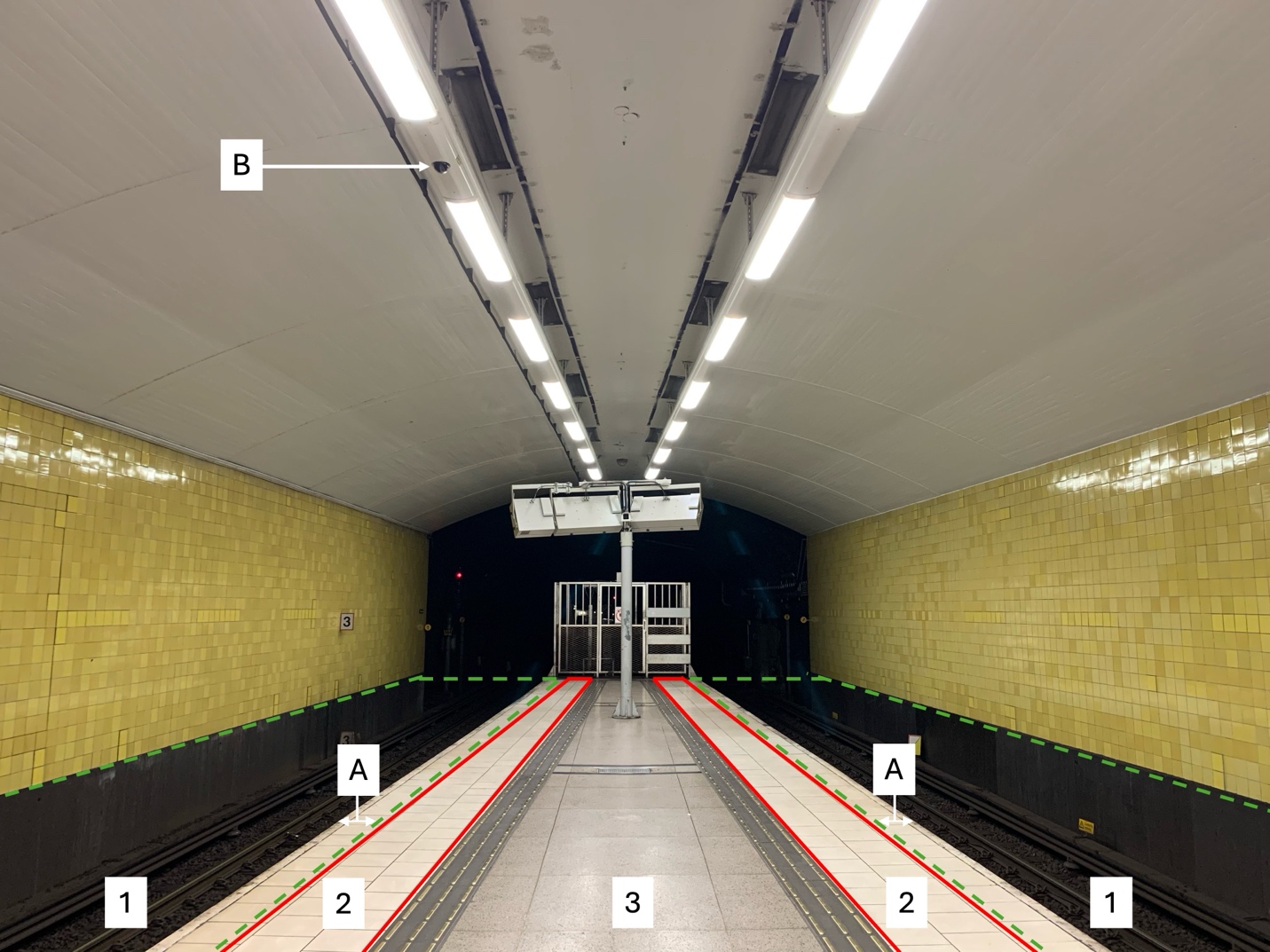


**Fig. S2.** Principle illustration of the practical consequences of how the system is structured (Fig. S1) is illustrated in this picture. In all versions of the AI-CCTV system, the position and size of an individual is determined by a rectangle surrounding it. The first version of the AI-CCTV system triggered an alarm 5 seconds after at least 70 % of the rectangle surrounding an individual entered Area 1. In a subsequent system update, additional alarm criteria were introduced, whereby an alarm was triggered when the center and one corner of the baseline (the lowest side of the rectangle) of this rectangle are within Area 2 for more than 20 seconds or in the foot area of a bench in Area 3 for more than 20 minutes. The distance from the platform edge required to trigger an alarm (A) depended on camera placement (B) and platform curvature at each station, ranging from 0 to ≈0.5 metres.

### Information for travellers about the system

There has not been any information at the stations about the operation of the specific AI‑CCTV system. All stations do, however, display general notices informing travellers that CCTV surveillance is in use. The only publicly available information regarding which stations have the AI‑CCTV system operating has been provided on the SL webpage.

## SL’s other measures for suicide prevention in the metro

Information in this section is based on personal communication with Mikaela Pettersson at SL, except where a published source is referenced. The public transporter in Stockholm (SL) is actively working with several new measures to prevent suicides and accidents on the tracks. As noted in Table S1, all interventions except the Blue LED lights were absent during the post-period primarily evaluated herein. Any eventual effects of the Blue LED-lights on the primary post-period analysis were deemed to be minimal, as the sample size was negligible.

| **Table S1.** New measures SL has tested to prevent suicides and accidents on the tracks in the Stockhom metro. | | | |
| --- | --- | --- | --- |
| **Variables** | | **Start** | **End (inclusive)** |
| *Confounders* | |  |  |
|  | Helpline signage (all stations) | Q1 2024 | Q1 2025 |
|  | Enhanced security information (8 stations) | Q1 2024 | Q2 2025 |
|  | Blue LED-lights (2 stations)* | Q2 2022 | Q2 2022 |
| *The lights were switched on for about 3 weeks during Q2 2022. | | | |

### Helpline signage

A helpline sign intervention was implemented at all 100 metro stations from January 2024 to March 2025 as part of a pilot study conducted by SL. Helpline signs were installed on every information board at the platforms (Fig. S3). There was at least one sign per platform, with larger stations having two.


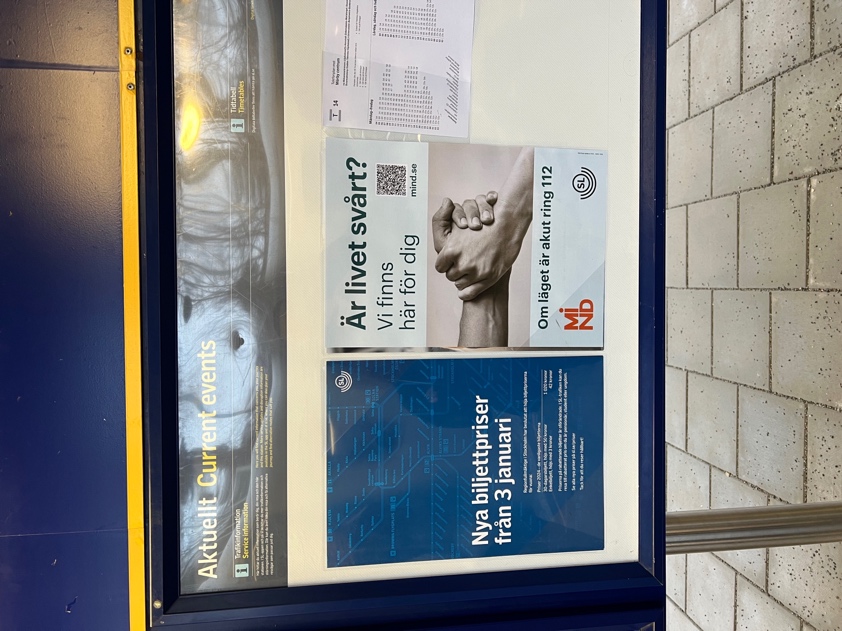


**Fig. S3.** Design of the sign informing about the helpline on an information board located at a metro platform.

### Enhanced Security Information

An “Enhanced Security Information” intervention was implemented at eight metro stations from mid-December 2023 as part of a pilot study conducted by SL. The purpose of the intervention was to clearly communicate how to act safely as a passenger at metro platforms and to restrict people from the beginning and end of every platform, since these parts of the platform are the areas where most suicides occur by jumping in front of a train or accessing the track area. This was achieved by adding an expanded white zone at the beginning and end of the platform, a yellow line marking the white line across the platform (Fig. S4), and installing signs indicating how to behave with respect to the yellow line (Fig. S5). This intervention was made to reduce suicides and trespass due to other motives. It is not anticipated that the effect of this intervention could be large, since it does not physically restrict anyone from jumping or assessing the tracks.


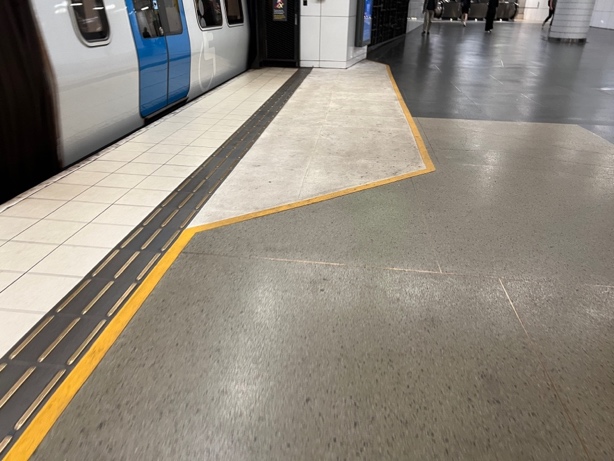


**Fig. S4.** Yellow line marking the white line and expanded white line in the beginning/end of the platform.


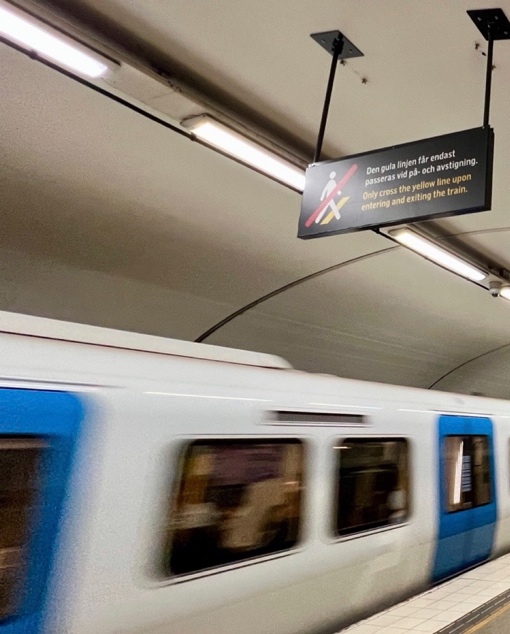


**Fig. S5.** Sign informing of how to behave in relation to the yellow line.

### Blue LED-lights

Blue LED lights were installed at two metro stations and turned on and off for a few weeks from May 2022 to June 2022 as part of a pilot conducted by SL. This pilot study was influenced by the results from a study in Japan reporting a reduction in suicides [3, 4]. The blue LED lights were installed at one underground (Fig S6) and one outdoor station (Fig. S7), located at the beginning of the platforms, where the risk of suicidal events is the highest. As these two metro stations were found in the control group, the effect of this exposure could not have contributed to AI-CCTV ITS effect in our analysis. One PUT due to suicidality occurred at a control station during the few weeks the blue LED lights were turned on, but this occurred outside during daylight, while the blue light was only noticeable directly under the lamp. We therefore concluded that this exposure played no role in CITS estimate of the primary analysis either.


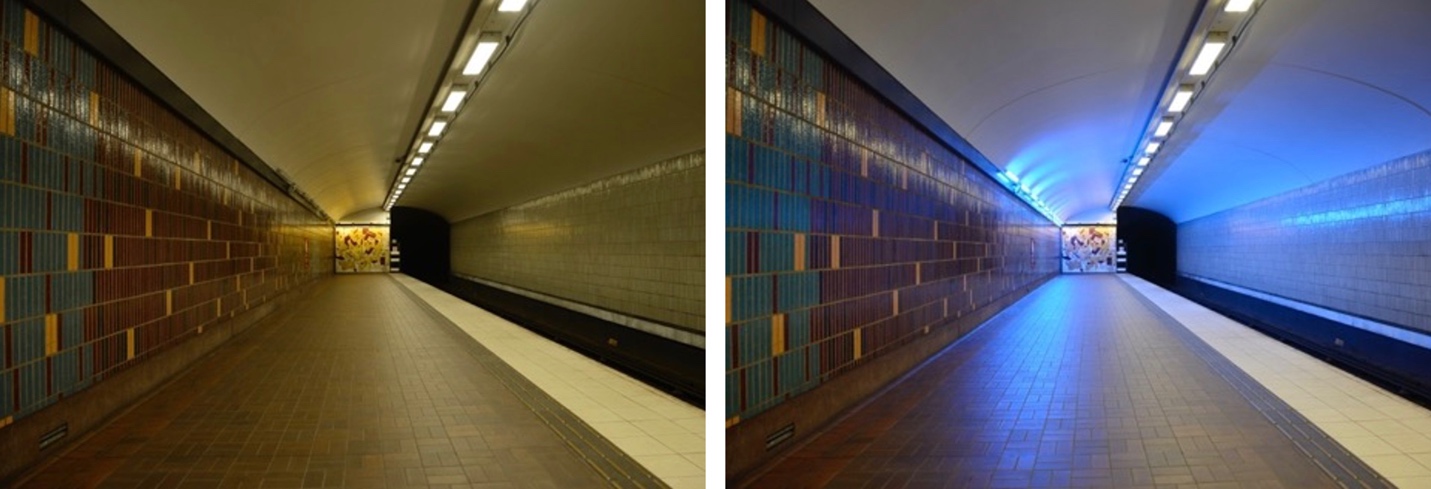


**Fig S6.** Blue LED-light at the underground station.


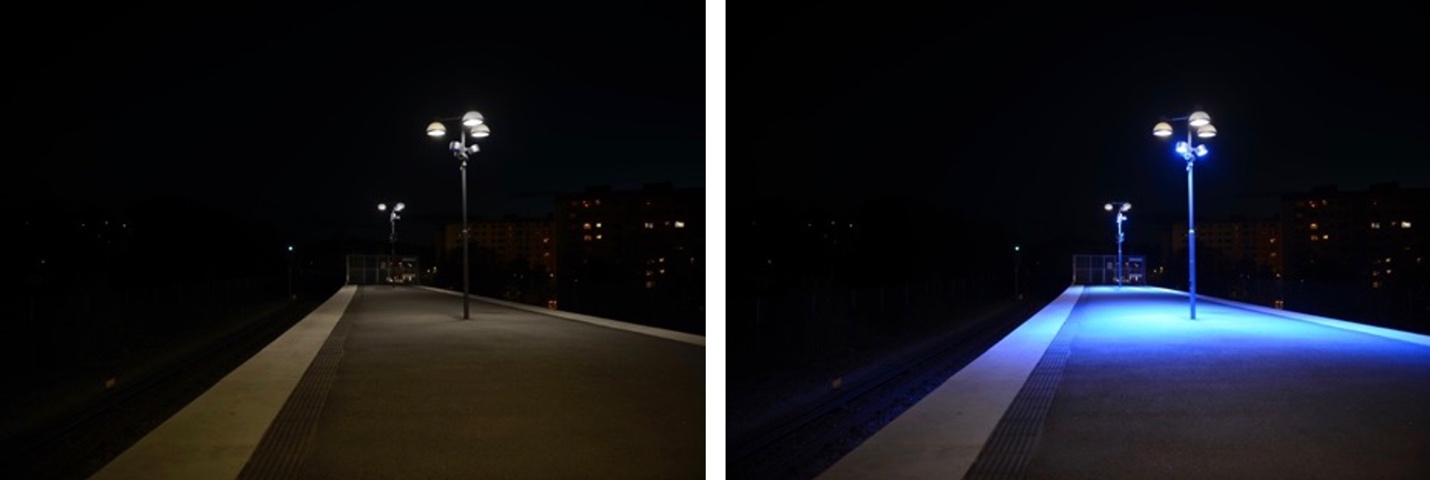


**Fig. S7.** Blue LED-light at the outdoor station

## Map of interventions


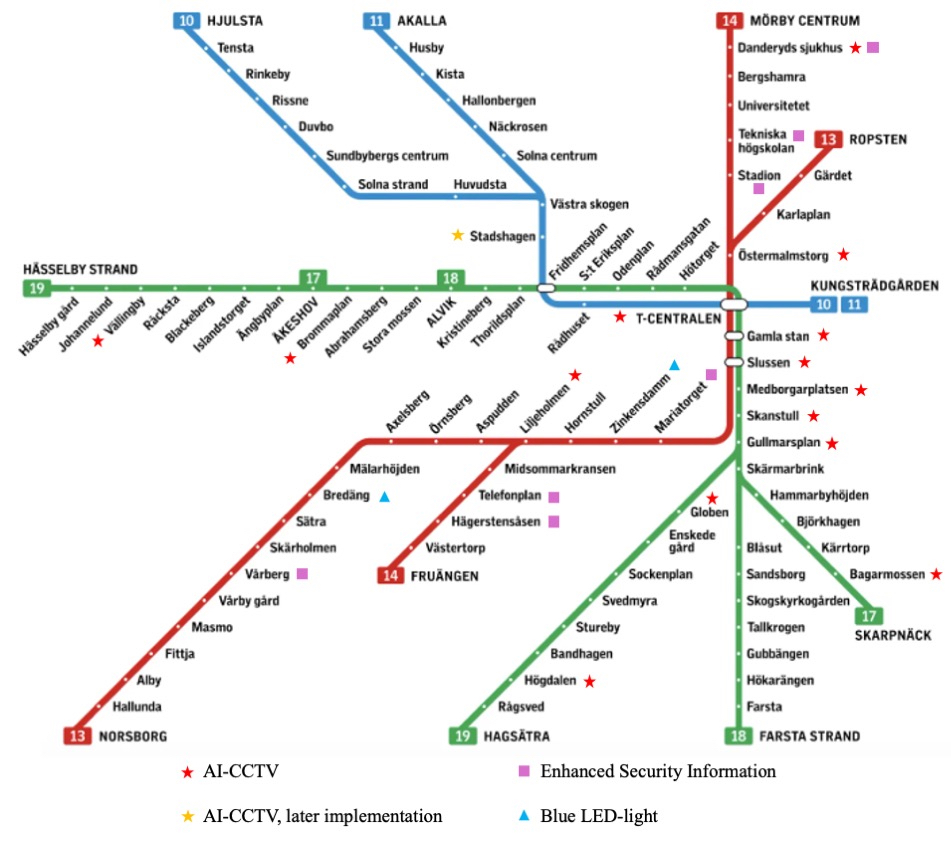


**Fig. S8.** The Stockholm Metro Network map that highlights the suicide preventive measures currently in use.

## The Stockholm Metro system characteristics

Information in this section is based on personal communication with Mikaela Pettersson at SL (January 2026), except where a published source is referenced. Stockholm County, home to Sweden’s capital city, has a population of about 2.5 million people, making it the country’s largest metropolitan region [5]. Key characteristics of the Stockholm metro are presented in Table S2.

| **Table S2.** The Stockholm Metro system characteristics | |
| --- | --- |
| **Attribute** | **Value** |
| *General* | |
| Owner | Region Stockholm Public Transporter, public name “SL”. |
| Operator of trains and stations | Regularly procured; from 2009 until 2025 by MTR. Since 2025 by Connecting Stockholm. |
| Network length | ≈110 km |
| Stations | 100 (47 underground, 53 above ground) |
| Metro lines and route numbers | Blue Line routes 10–11, Red Line routes 13–14, Green Line routes 17–19.^a^ |
| Travellers | In 2022, there were ≈900,000 trips on a typical winter weekday; fewer during summer and on weekends. |
| Opening years | 1950 (Green), 1964 (Red), 1975 (Blue) |
| Rolling stock | SL C20 (3-car sets) and SL C30 (2-car sets). Until 2024, SL CX (8-car sets) were also in use. |
| Speed limit between stations | 70 km/h on the green line, 80 km/h on the Red and Blue line |
| Speed limit at stations | 50 km/h when entering a station. |
| Track and power | Ballasted track; third rail (650–750 V DC) |
| Ridership | ≈330 million trips/year (~0.9 million/day) |
| Peak frequency | ≈2 min headways (up to 30 trains/hour per direction on busiest sections) on shared sections of the Green line; ≈2.5 min headways on shared sections of the Red and Blue lines; ≈5 min headways on suburban sections |
| Off-peak daytime frequency | ≈3 min headways on shared sections of the Green line; ≈5 min headways on shared sections of the Red and Blue lines; ≈10 min headways on suburban sections |
| Evening frequency | ≈15 min on suburban sections |
| Late night frequency | ≈30 min on suburban sections |
| Opening hours | ≈5 am to ≈1 am on weekdays; open 24 h on weekends |
|  |  |
| *Safety and security measures relevant for suicide prevention and PUT accidents* | |
| Fencing between stations | Yes; all sections between stations are “intrusion safe” either by tunnels, bridges, or fencing. Fences are at least ≈2.0 m high with barbed wire on top. |
| Platform screen doors | No |
| Emergency buttons to stop trains | Available only onboard trains, not on platforms |
| “Suicide pits” | No, only ballasted tracks |
| Safety and security hotline for travellers | Yes; accessible via travellers’ own mobile telephone or chat. No landline phones on the platforms. |
| Suicide prevention programme | The Stockholm Region has had a suicide prevention strategy since 15 May 2020. SL has an action plan for suicide prevention in public transport since 27 Jan 2025. |
|  |  |
| ^a^ The Stockholm metro consists of three main lines, each in a different color. The numbers refer to route numbers within each line. This is also shown in the metro map in Figure S8. | |

## AI-CCTV media attention

SL has frequently participated in and presented the AI-CCTV in national and regional mass media since September 27, 2022, and has continued to do so. An unsystematic search, confirmed with SL regarding media reports about AI-CCTV, is presented in Table S3.

| **Table S3.** Examples of media attention of AI-CCTV | | | |
| --- | --- | --- | --- |
| **Outlet/Format [in Swedish]** | **Title (Swedish) [English translation]** | **AI-CCTV Stations mentioned?** | **Publication date** |
| Dagens Nyheter – News article [in Swedish] | [Ny AI-teknik ska minska självmord i SL-trafiken [New AI technology aims to reduce suicides in Stockholm public transport]](https://www.dn.se/sverige/ny-ai-teknik-ska-minska-sjalvmord-i-sl-trafiken/) | No | 2022-09-27 |
| TV4 – News segment – Efter Fem [in Swedish] | [AI ska förhindra självmord i tunnelbanetrafiken [AI to prevent suicides in the metro]](https://www.tv4play.se/klipp/6b73af8a8e2a46b0a824?first=13760888&playlist=52Wgt5XxjuRP6j4hkDRKpz&offset=0&section=efter-fem) | No | 2022-09-29 |
| Mitt i – News article [in Swedish] | [AI har räddat 17 liv i tunnelbanan [AI has saved 17 lives in the metro]](https://www.mitti.se/nyheter/ai-har-raddat-17-liv-i-tunnelbanan-6.3.208097.1a5fa2ab1f) | Yes | 2024-03-05 |
| Dagens Nyheter – News article [in Swedish] | [AI räddar liv i tunnelbanan: 17 dödsfall tros ha förhindrats [AI saves lives in the metro: 17 deaths believed to have been prevented]](https://www.dn.se/sverige/ai-raddar-liv-i-tunnelbanan-17-dodsfall-tros-ha-forhindrats/) | Yes | 2024-03-18 |
| TV4 – News segment – Efter Fem [in Swedish] | [AI-teknik räddar liv i Stockholms tunnelbana [AI technology saves lives in Stockholm's metro]](https://www.tv4play.se/klipp/5e418882012df4a60993/video-ai-teknik-raddar-liv-i-stockholms-tunnelbana-21-dodsfall-tros-ha-forhindrats) | No | 2024-03-21 |
| Mina drömmars stad – Podcast on Stockholm local politics [in Swedish] | [Mina drömmars stad? #20: AI-övervakad i tuben [My Dream City? #20: AI-surveilled in the metro]](https://open.spotify.com/episode/0DwvSigVk9h4Cp0uVqzKfD?si=eObNX3bIQQOel7Rar_4xTw&t=1026&context=spotify%3Ashow%3A2FGvKTUKJVZS3R7x6XBMZm&nd=1&dlsi=469aaf8fd6dc48d3) | No | 2024-04-01 |
| SVT – In-depth TV program – Vetenskapens Värld [in Swedish] | [AI på liv och död [AI: A matter of life and death]](https://www.svtplay.se/video/KnDgPgm/ai-pa-liv-och-dod) | No | 2024-08-25 |
| Sveriges Radio P4 Stockholm – Radio segment [in Swedish] | [AI bakom fortsatt livräddande framgångar i tunnelbanan [AI behind continued life-saving successes in the metro]](https://www.sverigesradio.se/artikel/ai-bakom-fortsatt-livraddande-framgangar-i-tunnelbanan) | Yes (15 stations) | 2024-11-29 |
| Järnvägsnyheter – News article [in Swedish] | [Plattformsdörrar på Gul linje – miljardbeslut i Stockholm [Platform screen doors on the Yellow line – billion-krona decision in Stockholm]](https://www.jarnvagsnyheter.se/20250331/17307/plattformsdorrar-pa-gul-linje-miljardbeslut-i-stockholm) | Yes (Yellow line) | 2025-03-31 |
| Svenska Dagbladet – News article [in Swedish] | [Självmord i tunnelbanan – så ska det förhindras [Suicide in the metro – how it will be prevented]](https://www.svd.se/a/Xj0JKg/sjalvmord-suicid-i-tunnelbanan-sa-ska-det-forhindras) | Yes (Vällingby; notes 15 stations) | 2025-06-09 |
| Breakit – News article [in Swedish] | [AI stoppar suicid i tunnelbanan: "30 förhindrade suicidförsök" [AI stops suicides in the metro: "30 prevented attempts"]](https://www.breakit.se/artikel/43453/ai-stoppar-suicid-i-tunnelbanan-30-forhindrade-suicidforsok) | Yes (15 stations) | 2025-06-09 |
| Omni – News brief [in Swedish] | [AI ska stoppa suicid i Stockholms tunnelbana [AI to stop suicides in Stockholm’s metro]](https://omni.se/a/wgz1JL) | Yes (15 stations) | 2025-06-09 |
| Aktuell Säkerhet – News article [in Swedish] | [AI-teknik stoppar självmordsförsök i tunnelbanan [AI technology stops suicide attempts in the metro]](https://www.aktuellsakerhet.se/ai-teknik-stoppar-sjalvmordsforsok-i-tunnelbanan/) | Yes (15 stations) | 2025-06-10 |

# Supplementary methods

## STROBE statement

| Table S4. STROBE Statement—Checklist of items that should be included in reports of *cohort studies* | | | |
| --- | --- | --- | --- |
|  | Item No | Recommendation | Manuscript (Yes, No, N/A), page |
| **Title and abstract** | 1 | (*a*) Indicate the study’s design with a commonly used term in the title or the abstract | Yes |
|  |  | (*b*) Provide in the abstract an informative and balanced summary of what was done and what was found | Yes |
| Introduction | | |  |
| Background/rationale | 2 | Explain the scientific background and rationale for the investigation being reported | Yes, 1-2 |
| Objectives | 3 | State specific objectives, including any prespecified hypotheses | Yes, 2 |
| Methods | | |  |
| Study design | 4 | Present key elements of study design early in the paper | Yes, 3-8 |
| Setting | 5 | Describe the setting, locations, and relevant dates, including periods of recruitment, exposure, follow-up, and data collection | Yes, 3-8 |
| Participants | 6 | (*a*) Give the eligibility criteria, and the sources and methods of selection of participants. Describe methods of follow-up | Yes, 3-8 |
|  |  | (*b*) For matched studies, give matching criteria and number of exposed and unexposed | N/A |
| Variables | 7 | Clearly define all outcomes, exposures, predictors, potential confounders, and effect modifiers. Give diagnostic criteria, if applicable | Yes, 3-8 |
| Data sources/ measurement | 8* | For each variable of interest, give sources of data and details of methods of assessment (measurement). Describe comparability of assessment methods if there is more than one group | Yes, 3-8 |
| Bias | 9 | Describe any efforts to address potential sources of bias | Yes, 3-8 |
| Study size | 10 | Explain how the study size was arrived at | Yes, 3-8 |
| Quantitative variables | 11 | Explain how quantitative variables were handled in the analyses. If applicable, describe which groupings were chosen and why | Yes, 3-8 |
| Statistical methods | 12 | (*a*) Describe all statistical methods, including those used to control for confounding | Yes, 3-8 |
|  |  | (*b*) Describe any methods used to examine subgroups and interactions | Yes, 3-8 |
|  |  | (*c*) Explain how missing data were addressed | N/A |
|  |  | (*d*) If applicable, explain how loss to follow-up was addressed | N/A |
|  |  | (*e*) Describe any sensitivity analyses | Yes, 3-8 |
| Results | | |  |
| Participants | 13* | (a) Report numbers of individuals at each stage of study—eg numbers potentially eligible, examined for eligibility, confirmed eligible, included in the study, completing follow-up, and analysed | Yes, 3-8 |
|  |  | (b) Give reasons for non-participation at each stage | N/A |
|  |  | (c) Consider use of a flow diagram | N/A |
| Descriptive data | 14* | (a) Give characteristics of study participants (eg demographic, clinical, social) and information on exposures and potential confounders | N/A |
|  |  | (b) Indicate number of participants with missing data for each variable of interest | N/A |
|  |  | (c) Summarise follow-up time (eg, average and total amount) | Yes, 8 |
| Outcome data | 15* | Report numbers of outcome events or summary measures over time | Yes, 8-9 |
| Main results | 16 | (*a*) Give unadjusted estimates and, if applicable, confounder-adjusted estimates and their precision (eg, 95% confidence interval). Make clear which confounders were adjusted for and why they were included | Yes, 8-9 |
|  |  | (*b*) Report category boundaries when continuous variables were categorized | Yes, 8 |
|  |  | (*c*) If relevant, consider translating estimates of relative risk into absolute risk for a meaningful time period | N/A |
| Other analyses | 17 | Report other analyses done—eg analyses of subgroups and interactions, and sensitivity analyses | Yes, 10-14 |
| Discussion | | |  |
| Key results | 18 | Summarise key results with reference to study objectives | Yes |
| Limitations | 19 | Discuss limitations of the study, taking into account sources of potential bias or imprecision. Discuss both direction and magnitude of any potential bias | Yes, 15-18 |
| Interpretation | 20 | Give a cautious overall interpretation of results considering objectives, limitations, multiplicity of analyses, results from similar studies, and other relevant evidence | Yes, 15-18 |
| Generalisability | 21 | Discuss the generalisability (external validity) of the study results | Yes, 16, 18 |
| Other information | | |  |
| Funding | 22 | Give the source of funding and the role of the funders for the present study and, if applicable, for the original study on which the present article is based | Yes, 20 |

*Give information separately for exposed and unexposed groups

## Classification of PUT incidents

This description of the classification process is based on personal communication with Mikaela Pettersson, SL.

The video films from all PUT incidents were reviewed to determine whether the event was related to suicidality or an accident. Classification was based on the available video material and contextual information from the incident. The assessment considered observed behavior before the event, the circumstances of the incident, and other available operational information.

The classification followed established principles used in routine incident review, but some degree of professional judgement was required, particularly in ambiguous cases. Most cases were reviewed by the same experienced staff member, who assessed approximately 95% of the material. A small number of other staff members could secure or initially review video material. Unclear cases were discussed jointly to support consistent classification. Most cases were considered clear, while only a small proportion required additional discussion.

This information was then complemented with a systematic classification procedure which also involved other entities not related to the metro operator, such as forensic / accident investigators and the police [6].

## Statistical analysis

### Pre-intervention diagnostics

Parallel trends in the pre-intervention period were assessed using a Poisson GLM with a group-by-time interaction term. No evidence of differential pre-intervention trends between intervention and control stations was observed (interaction *IRR* 1.01, 95% CI 0.98–1.03; *p* = 0.52).

### Dispersion

#### CITS

For the controlled Poisson GLM, diagnostic statistics indicated overdispersion and with large discrepancy between the deviance (4.1) and Pearson (895) dispersion statistics. Inspecting residuals showed that the highly inflated Pearson statistic was mainly due to four outlier values occurring when the counts where >0 for the AI-stations group, while being offset using 0.001 values for the control group. Poisson regression with robust standard errors was retained to obtain valid confidence intervals and *p*-values.

#### ITS

For the Poisson GLM, diagnostic statistics indicated mild underdispersion, with a Pearson dispersion statistic of 0.83 and a deviance dispersion statistic of 0.99. Poisson regression with robust standard errors was retained.

### Autocorrelation and stationarity

Residual autocorrelation in the primary CITS model was assessed using Ljung–Box tests on deviance residuals, conducted separately for intervention and control stations. No evidence of residual autocorrelation was detected at lag 4 (Q = 3.95, p = 0.41). In addition, deviance residuals showed a white noise pattern using Bartlett’s periodogram-based white-noise test as well as in simple plots against time.

### Seasonal trends

Seasonality was examined by including a dummy variable for season (1–4), given prior evidence of seasonal variation in suicide events in the Stockholm Metro [7].

### E value

The E-value calculator was used at <https://www.evalue-calculator.com/> [8] for the statistical interaction CITS estimate. The E-value was 5.7 for the primary CITS point estimate (*IRR* = 0.27) and 1.81 for the robust 90% confidence interval limit closest to the null (one-tailed 90% CI upper = 0.80). This indicates confounder would need have moderate to strong association with both AI-CCTV implementation and changes in PUT due to suicidality in a similarly timed manner, to fully explain the observed association [9]. These E-values of 5.7 and 1.81 were well above the median of many other epidemiological studies (2.00 and 1.41) [10]. Clearly, the confidence interval limit was more sensitive than the point estimate, to a putative confounder able to cause both the AI-CCTV implementation and a reduction in PUT due to suicidality, during the same time periods, but we found no plausible theory as to how that could realistically occur. The spatiotemporal details about the AI-CCTV implementation were known only by a subset of mainly technical or other SL transport staff insiders during the first year, professionals who are not involved in e.g. mental health care or otherwise in contact with suicidal subjects.

### Sensitivity analyses

Prespecified sensitivity analyses are described in the article; implementation details and summary results are provided in Table S5.

#### Negative binomial distribution

We refitted the primary CITS using a negative binomial variance function (log link) to relax the Poisson equidispersion assumption.

#### Omitting seasonal terms

We refitted the primary CITS, excluding seasonal indicator terms.

#### Exposure fidelity

We refitted the primary CITS, excluding the single event that occurred despite AI-CCTV being in place, due to insufficient image resolution and/or limited camera field of view (Table S8).

#### Placebo breakpoint

We refitted the primary CITS with a false breakpoint placed at the median of the pre-intervention period.

Intervention only ITS, omitting seasonal terms
We fitted an ITS model for the intervention sites only, excluding seasonal indicator terms.

#### Benchmark models

We fitted a difference-in-differences model and a pre-post model for intervention sites only, without adjusting for underlying time trends.

#### Displacement effects to nearby control stations

If there is a displacement effect from intervention stations to nearby control stations, we hypothesize that control stations in closer proximity to intervention stations would be more affected than those farther away. We examined this by comparing control stations one stop from an AI-CCTV station with control stations two or more stops away. We aggregated the number of events within each distance group for the pre-intervention and post-intervention periods and constructed a 2×2 contingency table with distance group and period as factors. The odds ratio quantifies whether the pre- to post-change differed between distance groups, and Fisher’s exact test was used because of small cell counts. For transparency, we also report the numbers of stations and events by distance category and period, as well as the period lengths (Table S6).

#### Bayesian sensitivity analysis using weak priors

We repeated the analyses with Bayesian inference using weakly informative priors, to mitigate putative sparse-data problems with the models and to gain further verification of the direction and magnitude of the effect. For epidemiological studies, odds ratio effects in the range of 0.1 – 10 have been recommended as a weak prior [11]. However, effects of means restriction against suicidality usually show stronger effects, e.g. *IRR* = 0.09 (95% CI 0.04-0.21) reported in a recent meta-analysis [12]. As AI-CCTV also is a form of means restriction (by stopping of trains after an AI-CCTV alarm is triggered), using *IRR* range of 0.1 – 10 is likely to limiting. We therefore also tested *IRR* 95% normal ranges of 0.05-20, 0.01-100, as well as the default, very weak prior in Stata (100 standard deviations, corresponding to *e*^(100*1.96)^ → 10^-85^ - 10^85^ 95% *IRR* interval). The prior mean was the default ln(1) = 0 on the log scale, i.e. *IRR*=1 for no effect. We used four chains of Markov Chain Monte Carlo (MCMC) simulations, each with N=10000 sample size. A maximum Gelman-Rubin convergence < 1.01, effective sample size (ESS) > 1000, no autocorrelation warnings and visually overlapping trace/histogram/density plot for the chains, were considered sufficiently reliable for Bayesian estimation. The posterior probability (*IRR* <1 | data) results reported are naturally one-tailed and the probability that there was an effect is here also referred to as: <0.8, insufficient to low probability; 0.8 – 0.9, moderate probability; 0.9 – 0.95, moderately high probability; >0.95, high probability.

## The extended post-intervention period

Media exposure has been shown to affect suicidal ideation [13] and several studies have documented associations between media reporting and suicide rates. In the Vienna metro, the introduction of media guidelines promoting responsible reporting about metro suicides was followed by a reduction in suicides [14], whereas periods of intensive media reporting were associated with higher metro suicide rates [15]. Similar findings have been reported from the Toronto metro, where each additional metro-related media article was associated with an approximate 2% increase in the suicide rate [16]. Research on the implementation of a suicide barrier at the Bloor Street Viaduct in Canada initially observed increased suicides at nearby bridges, a pattern that decreased over time and was attributed to changes in media reporting intensity [17]. From Q4 2022, the AI-CCTV intervention received increasingly intensified media coverage (Table S3), which represents a concurrent external event and increases the risk of bias by influencing suicidal behaviour in unpredictable ways. To support causal inference, the primary analyses therefore focused on the post-intervention period preceding the intensified media attention. However, an exploratory extension beyond the primary post-intervention (from Q4 2022 to 2025Q2 is presented). This period encompassed substantial contextual changes, including increased media attention, modifications to the AI-CCTV system, additional suicide-prevention measures within the Stockholm metro, and the implementation of AI-CCTV at one additional station being in the control group (with *n*=6 PUT in the pre-period, none in the post-periods). To avoid exposure misclassification, that station was now excluded from the control group. Results from this extended period are exploratory and not intended for causal inference about the effect of AI-CCTV exposure on the primary outcome.

## Statistical analysis of secondary outcomes

Secondary outcomes were analysed using simplified quasi-experimental comparisons for outcomes available at the station–quarter level; pre–post comparisons; and difference-in-differences analyses, conducted using Poisson regression to contrast changes at stations with AI-CCTV with those at control stations. All secondary analyses were restricted to the direct post-implementation period to assess immediate effects.

Outcomes with very low event counts, including suicide due to PUT, were analysed using exact Poisson regression, with limited statistical power. Descriptive summaries are shown for AI-CCTV alarms and PUT occurring despite AI-CCTV. Train-traffic cancellations were analysed as continuous outcomes using pre–post and difference-in-differences comparisons. Results for all secondary outcomes are reported in the Supplementary Results.

# Supplementary results

## Primary outcome: additional analyses

The robustness of the findings was assessed through a set of prespecified sensitivity analyses (Table S5). Results were consistent across alternative specifications and did not materially contradict the primary results.

| **Table S5.** Sensitivity analysis | |
| --- | --- |
| **CITS sensitivity analyses** | **Effect estimate** |
| CITS with negative binomial distribution | *IRR* 0.12, 90% CI 0.02 to 0.90, one-sided *p*=0.042 |
| CITS omitting seasonal terms | *IRR* 0.26, 90% CI 0.11 to 0.66, one-sided *p*=0.009 |
| CITS accounting for exposure fidelity | *IRR* = 0.16, 90% CI 0.04 to 0.57; one-sided *p* = 0.009 |
| Pseudo-intervention CITS (median pre-intervention breakpoint) | *IRR* = 2.12, 90% CI 0.57 to 7.83; one-sided *p* = 0.17 |
|  |  |
| **Alternative time series specification** |  |
| ITS omitting seasonal terms (intervention only) | *IRR* = 0.41, 90% CI 0.16 to 1.07; one-sided *p* = 0.064 |
|  |  |
| **Simplified benchmark models** |  |
| Difference-in-Differences | *IRR* = 0.33, 90% CI 0.15 to 0.72; one-sided *p* = 0.035 |
| Pre-post (intervention only) | *IRR* = 0.43, 90% CI 0.18 to 1.00; one-sided *p* = 0.0505 |
|  | |
| **Additional sensitivity analyses** |  |
| Displacement effects: adjacent vs non adjacent control stations | *OR*=0.91, 95% CI 0.15 to 3.98; two-sided Fisher’s exact *p* = 1.00 |
| *Note.* Estimates are reported as IRRs from log link models. The primary and most sensitivity models used Poisson GLMs with robust standard errors; the overdispersion sensitivity used a negative binomial model with the same CITS specification. CITS models include level terms. The placebo analysis used the same CITS specification with a false breakpoint at the median of the pre-intervention period. Difference-in-differences and pre–post models are simplified benchmarks and do not adjust for underlying time trends. Transfer effects were assessed using a 2×2 table crossing period (pre vs post) and proximity group (adjacent control stations defined as one stop from an intervention station vs non adjacent defined as two or more stops), based on aggregated event counts; Fisher’s exact test was used due to small cell counts. Similar result were found for station 1-2 stops or 1-3 stops away from the AI-CCTV stations (not shown). | |

| **Table S6.** Event counts by distance to intervention stations | | | | | | | |
| --- | --- | --- | --- | --- | --- | --- | --- |
|  | **Station with AI-CCTV** | **One stop away** | **Two stops away** | **Three stops away** | **Four stops away** | **Five stops away** | **Six and more stops away** |
| Number of stations | 14 | 21 | 19 | 9 | 6 | 5 | 26 |
| Number of events, pre | 55 | 29 | 15 | 14 | 8 | 5 | 37 |
| Number of events, post | 2 | 3 | 1 | 3 | 1 | 1 | 3 |
| *Note.* The “Station with AI-CCTV” column refers to intervention stations. Control stations are grouped by the minimum stop distance to the nearest intervention station. The pre-intervention period comprised 46 quarters and the post intervention period 4 quarters; within each period, all stations contributed the same number of quarters. The *OR* analysis of displacement effects (Table S5) compared control stations one stop away versus two or more stops away (all ≥2 stops combined); intervention stations were excluded from that analysis. | | | | | | | |

## Secondary outcomes

Secondary outcomes are presented to provide complementary information relevant to interpreting the primary analysis and to describe potential mechanisms and system-level effects of AI-CCTV. Given lower event counts, secondary outcomes should be interpreted cautiously and are intended to provide contextual rather than definitive causal information. An exception is death by suicide, which was prespecified as a key secondary outcome and constitutes a subset of the primary outcome. For this outcome, effect estimates are interpreted causally within the same controlled interrupted time-series framework as in the primary analysis.

### Time periods for secondary outcome

Time periods for the respective secondary outcomes are presented in Table 1 in the article.

### Secondary outcome analysis

#### Death by suicide

Deaths by suicide at stations with AI-CCTV decreased to zero in the post-implementation period, while six suicides occurred at control stations (Table S7).

#### Individuals safeguarded

Data on the number of individuals safeguarded due to suicidality were provided by SL and analysed as an intermediate outcome reflecting overall safeguarding activity (Table S7). This measure is broader than PUT and does not specifically capture the most acute high-risk situations at the platform edge. Events representing the highest risk level are instead described under the sections AI-CCTV alarms and operational responses, and PUT despite AI-CCTV.

#### PUT due to accidents

PUT due to accidents showed point estimates consistent with a reduction following implementation (Table S7), although uncertainty was substantial due to low outcome counts.

#### Train traffic cancellations

Data on cancelled train-kilometres due to person-under-train events and trespassing were provided by SL and analysed as system-level outcomes. Analyses were restricted to the direct post-implementation period to assess immediate effects. Cancelled train-kilometres capture both cancelled services and, indirectly, also severe service delays, as major disruptions in the Stockholm metro system are typically managed through service cancellations.

| **Table S7.** Summary of secondary outcome analyses. | | | | |
| --- | --- | --- | --- | --- |
| **Outcome** | **Analysis** | **Estimate** | **90% CI**  **or**  ***R_c_* / ESS** | **One-sided *p*-value or Probability (*IRR* < 1 \| data)** |
| Death by suicide | Difference-in-differences | 0.31^1^ | 0.00 to 1.70^1^ | 0.140^1^ |
| Death by suicide | Difference-in-differences, Bayesian^2^ | 0.23^2^ | 1.001 / 6816 | 0.9382^2^ |
| Death by suicide | Pre-post | 0.30^1^ | 0 to 1.33^1^ | 0.102^1^ |
| Death by suicide | Pre-post, Bayesian^2^ | 0.21^2^ | 1.000 / 7796^2^ | 0.962425^2^ |
| Safeguarded individuals | Difference-in-differences | 0.79 | 0.57 to 1.08 | 0.108 |
| Safeguarded individuals | Pre-post | 0.79 | 0.64 to 0.98 | 0.0335 |
| PUT due to accidents | Difference-in-differences | 0.21^1^ | 0.01 to 1.80^1^ | 0.16^1^ |
| PUT due to accidents | Difference-in-differences, Bayesian^2^ | 0.32^2^ | 1.002 / 3642^2^ | 0.90815^2^ |
| PUT due to accidents | Pre-post | 0.49^1^ | 0.02 to 2.51^1^ | 0.41^1^ |
| PUT due to accidents | Pre-post, Bayesian^2^ | 0.52^2^ | 1.001 / 7844^2^ | 0.804875^2^ |
| Train cancellations | Difference-in-differences | -1509^3^ | -2612 to -405 | 0.012 |
| Train cancellations | Pre-post | -1610^3^ | -2265 to -956 | < 0.001 |
| *Note.* Estimates are reported with one sided 90% confidence intervals. All estimates compare post intervention period with the pre intervention period. Statistical inference was based on prespecified one sided tests. Difference-in-differences estimates provide the primary inference for secondary outcomes and are consistent with the controlled interrupted time series design. Pre-post estimates are reported for descriptive purposes only. Pre-post and difference-in-differences estimates were obtained using Poisson regression for count outcomes and linear regression for train cancellation outcomes, as specified in the Methods. P values for secondary outcomes are reported for completeness. No adjustment for multiple comparisons was applied.  ^1^ exact Poisson, due to low outcome counts  ^2^ Bayesian estimation using weakly informative priors, i.e. 95% interval 0.05-20 for *IRR.* Shown are the Bayesian 50% posterior median *IRR*, maximum Gelman–Rubin *R_c_* / the effective sample size (ESS) as well as the posterior Probability (*IRR* < 1 \| data).  ^3^ gaussian GLM | | | | |

#### AI-CCTV alarms and operational responses

Descriptive analyses were conducted across the full implementation period to characterise the operational capacity and functional performance of the AI-CCTV system. These analyses were not intended to assess temporal trends or intervention effects. All AI-CCTV alarms that resulted in operational responses and safeguarding of individuals were included. Responses involved measures such as train speed reduction, stopping of trains, or power disconnection. By Q2 2025, a total of 36 such events had been recorded, most occurring at the platform edge, with fewer in the track area. Detailed characteristics are reported in Table S8.

| **Table S8.** Characteristics of AI-CCTV alarms resulting in operational responses and safeguarding before a PUT. | | | | | | | |
| --- | --- | --- | --- | --- | --- | --- | --- |
| **Quarter** | **n** | **Station type (Underground/**  **Outdoor)** | **Station design (Open/**  **Partition wall)** | **Area (Inner city/Suburb)** | **Platform geometry (Straight/Curved/**  **Slightly curved)** | **AI-CCTV functionality (Initial/Extended)** | **AI-CCTV**  **trigger (Platform edge/Trespass)** |
| 2021Q4 | 2 | 0/2 | 2/0 | 0/2 | 1/1/0 | 2/0 | 2/0 |
| 2022Q1 | 0 | 0/0 | 0/0 | 0/0 | 0/0/0 | 0/0 | 0/0 |
| 2022Q2 | 5 | 4/1 | 4/1 | 3/2 | 1/3/1 | 5/0 | 4/1 |
| 2022Q3 | 0 | 0/0 | 0/0 | 0/0 | 0/0/0 | 0/0 | 0/0 |
| 2022Q4 | 3 | 2/1 | 3/0 | 3/0 | 1/0/2 | 3/0 | 2/1 |
| 2023Q1 | 3 | 3/0 | 1/2 | 3/0 | 2/1/0 | 3/0 | 0/3 |
| 2023Q2 | 1 | 1/0 | 1/0 | 1/0 | 1/0/0 | 1/0 | 1/0 |
| 2023Q3 | 2 | 2/0 | 2/0 | 1/1 | 1/0/1 | 0/2 | 2/0 |
| 2023Q4 | 3 | 3/0 | 0/3 | 3/0 | 3/0/0 | 0/3 | 2/1 |
| 2024Q1 | 2 | 1/1 | 1/1 | 1/1 | 1/1/0 | 0/2 | 0/2 |
| 2024Q2 | 4 | 2/2 | 3/1 | 2/2 | 3/1/0 | 0/4 | 4/0 |
| 2024Q3 | 4 | 4/0 | 3/1 | 4/0 | 2/2/0 | 0/4 | 4/0 |
| 2024Q4 | 4 | 4/0 | 3/1 | 4/0 | 1/3/0 | 0/4 | 2/2 |
| 2025Q1 | 1 | 1/0 | 1/0 | 1/0 | 0/1/0 | 0/1 | 0/1 |
| 2025Q2 | 2 | 2/0 | 2/0 | 2/0 | 1/1/0 | 0/2 | 0/2 |
| **Total** | **36** | **29/7** | **26/10** | **28/8** | **18/14/4** | **14/22** | **23/13** |

#### PUT despite AI-CCTV

Data on PUT occurring despite AI-CCTV after implementation was provided by SL and reviewed descriptively to identify recurring operational limitations and failure modes. All such events were included. Most were associated with late platform jumps immediately before train arrival or with technical limitations affecting detection. Case-level details are reported in Table S9.

| **Table S9.** PUT despite AI-CCTV after implementation. | | | | | | | |
| --- | --- | --- | --- | --- | --- | --- | --- |
| **Quarter** | **n** | **Station type (Underground/Outdoor)** | **Station design (Open/Partition)** | **Area (Inner/Suburb)** | **Platform geometry (Straight/Curved/Slightly curved)** | **AI-CCTV functionality (Initial/Extended)** | **Explanation** |
| 2022Q1 | 1 | 1/0 | 0/1 | 1/0 | 1/0/0 | 1/0 | Jumped from platform just before train arrival |
| 2022Q2 | 1 | 1/0 | 1/0 | 1/0 | 0/1/0 | 1/0 | Insufficient image resolution for detection despite presence on the tracks. |
| 2023Q1 | 1 | 0/1 | 1/0 | 1/0 | 0/0/1 | 0/1 | Jumped from platform just before train arrival |
| 2023Q2 | 3 | 1/2 | 3/0 | 1/2 | 1/1/1 | 0/3 | Jumped from platform just before train arrival |
| 2023Q3 | 2 | 2/0 | 2/0 | 1/1 | 0/0/2 | 0/2 | Jumped from platform just before train arrival |
| 2023Q4 | 1 | 0/1 | 1/0 | 0/1 | 0/1/0 | 0/1 | Insufficient image resolution for detection despite presence on the tracks |
| 2024Q2 | 1 | 1/0 | 0/1 | 1/0 | 1/0/0 | 0/1 | Insufficient image resolution for detection despite prolonged presence on the edge of the white line |
| 2025Q1 | 2 | 1/1 | 2/0 | 1/1 | 0/2/0 | 0/2 | Jumped from platform just before train arrival |
| **Total** | **12** | **7/5** | **10/2** | **7/5** | **3/5/4** | **2/10** | **9 Jumped from platform just before train arrival**  **3 Insufficient image resolution for detection** |
| *Quarters without cases are omitted. | | | | | | | |

## Exploratory analysis of the extended post-period

Media reporting about the AI-CCTV implementation began at the immediate end of Q3 2022 and then persisted continually going forward. Between Q1 2024 and Q1 2025, other interventions were also used in the metro system, e.g. a helpline signage for all stations and enhanced security information at 8 stations (one also having AI-CCTV). These additional exposures make it difficult to separate changes following AI-CCTV implementation from other concurrent influences. Combining the primary intervention period with the extended post-period, into one long and heterogeneous exposures post-period, rendered the above reported CITS level-change estimate no longer significant (*IRR* 0.54, 90% CI 0.22-1.34; one-sided *p*=0.133). When instead exploring ITS models specifying the two post-periods separately (Fig. S9A), levels of PUT due to suicidality at the AI-CCTV stations remained decreased for the primary post-period (*IRR* = 0.41, 90% CI 0.18-0.95; one-sided *p*=0.04), with a similar level-reduction at the end of the extended post-period in Q2 2025 (*IRR* = 0.42, one-sided *p*<0.1). In contrast, the same ITS model for the control stations did not indicate such a magnitude of reduction at the end of the extended post-period in Q2 2025 (*IRR* = 0.89, one-sided *p*>0.4). We next tested the CITS approach used in the main analysis, which is practical with two separate post-periods [18], results of which again supported the observed level-reductions in the primary post-period (*IRR* = 0.27, one-sided *p*<0.01), but less so at the end of the extended post-period in 2025 (*IRR* = 0.53, one-sided *p*>0.2). Thus, the primarily observed level change of AI-CCTV was retained in the context of using a longer time series, but the additional heterogeneous exposures in the extended post-period did not appear to strengthen the observed changes at the AI-CCTV stations *per se*. Finally, the systemic nature of exposures during the extended post-period (i.e., media reporting and helpline signage) had the potential to affect PUT due to suicidality in the entire metro system, i.e., including control stations. Running the ITS model using PUT due to suicidality in the entire metro system as outcome (Fig. S9B), indicated that there was a decreasing trend of about 5% less suicides for each quarter during the extended post-period (*IRR* = 0.95, one-sided *p*<0.05), which is thus an estimate of all heterogeneous exposures combined.


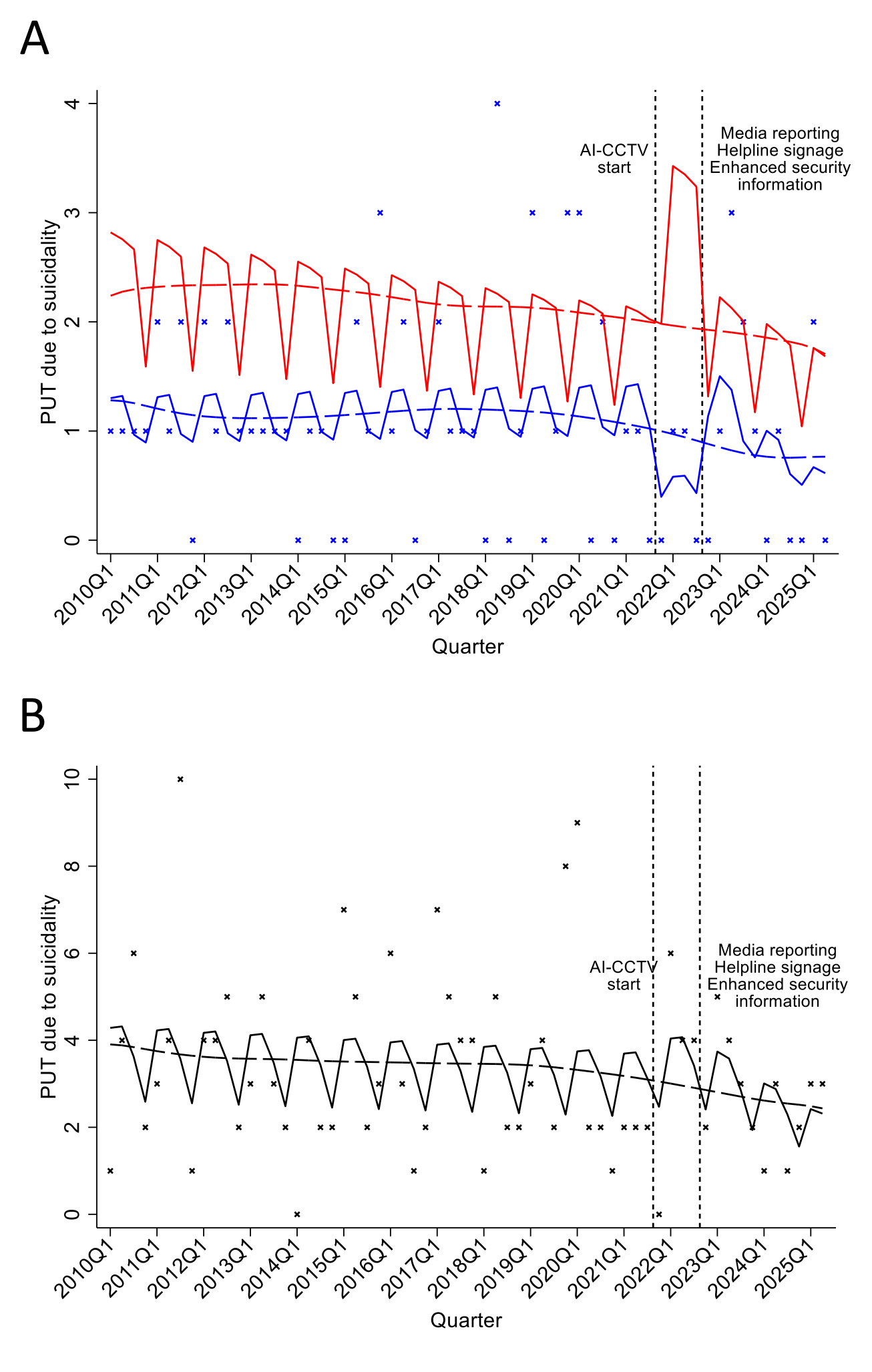


**Fig. S9. Exploring time series models including the extended post-period.** **(A)** Model with the primary level change estimate of the main Q4 2021- Q3 2022 period, as well as level and trend change estimates for the additional, extended post-period segment. Trend change was additionally modeled for the extended post-period, as media reporting was perpetual and the effects of helpline signages and yellow lines at station platforms may act gradually over time. Red line depict the ITS model for the control group (which acts as the counterfactual in CITS), while blue line depict the ITS model for the AI-CCTV stations group. Dashed lines depict LOESS trend curves of the quarterly PUT outcome counts at AI-CCTV intervention (blue x-marks) or control stations (not depicted). **(B)** ITS model of PUT due to suicidality among all 100 metro stations combined. Dashed line depicts the LOESS trend curve of the quarterly PUT outcome counts at all 100 metro stations (black x-marks).

# References

1. Irisity AB. AI That Saves Lives: Stockholm Metro’s Smart Surveillance is Changing Public Safety. Irisity. 2025. https://irisity.com/news-press/ai-surveillance-stockholm-metro/. Accessed 3 Sept 2025.

2. Irisity vinner SLs omfattande upphandling av videoanalys för tunnelbana. News Powered by Cision. 2020. https://news.cision.com/se/irisity-ab/r/irisity-vinner-sls-omfattande-upphandling-av-videoanalys-for-tunnelbana,c3245419. Accessed 15 Dec 2025.

3. Matsubayashi T, Sawada Y, Ueda M. Does the installation of blue lights on train platforms prevent suicide? A before-and-after observational study from Japan. Journal of Affective Disorders. 2013;147:385–8. https://doi.org/10.1016/j.jad.2012.08.018.

4. Matsubayashi T, Sawada Y, Ueda M. Does the installation of blue Lights on train platforms shift suicide to another station?: Evidence from Japan. Journal of Affective Disorders. 2014;169:57–60. https://doi.org/10.1016/j.jad.2014.07.036.

5. Statistics Sweden (SCB). Folkmängd i riket, län och kommuner 31 december 2024 och befolkningsförändringar 2024. Statistikmyndigheten SCB. https://www.scb.se/hitta-statistik/statistik-efter-amne/befolkning-och-levnadsforhallanden/befolkningens-sammansattning-och-utveckling/befolkningsstatistik/pong/tabell-och-diagram/folkmangd-och-befolkningsforandringar---helarsstatistik/folkmangd-i-riket-lan-och-kommuner-31-december-2024-och-befolkningsforandringar-2024/. Accessed 3 Sept 2025.

6. Andersson A-L, Liss G, Sokolowski M. Accident or suicide? New registration procedures and improved classification of suicide vs accident deaths on the Swedish railways: an interrupted time series analysis of years 2000–2023. Transportation Research Interdisciplinary Perspectives. 2025;34:101650. https://doi.org/10.1016/j.trip.2025.101650.

7. Uittenbogaard A, Ceccato V. Temporal and spatial patterns of suicides in Stockholm’s subway stations. Accident Analysis and Prevention. 2015;81:96–106. https://doi.org/10.1016/j.aap.2015.03.043.

8. Mathur MB, Ding P, Riddell CA, VanderWeele TJ. Web Site and R Package for Computing E-values. Epidemiology. 2018;29:e45. https://doi.org/10.1097/EDE.0000000000000864.

9. VanderWeele TJ, Ding P. Sensitivity Analysis in Observational Research: Introducing the E-Value. Ann Intern Med. 2017;167:268–74. https://doi.org/10.7326/M16-2607.

10. Trinquart L, Erlinger AL, Petersen JM, Fox M, Galea S. Applying the E Value to Assess the Robustness of Epidemiologic Fields of Inquiry to Unmeasured Confounding. American Journal of Epidemiology. 2019;188:1174–80. https://doi.org/10.1093/aje/kwz063.

11. Hamra GB, MacLehose RF, Cole SR. Sensitivity analyses for sparse-data problems-using weakly informative bayesian priors. Epidemiology. 2013;24:233–9. https://doi.org/10.1097/EDE.0b013e318280db1d.

12. Too LS, Shin S, Taouk Y, Pirkis J, Sinyor M, Yip PSF, et al. Impact of interventions at frequently used suicide locations on occurrence of suicides at other sites: a systematic review and meta-analysis. Psychol Med. 2025;55:e168. https://doi.org/10.1017/S0033291725100792.

13. Niederkrotenthaler T, Till B, Kirchner S, Sinyor M, Braun M, Pirkis J, et al. Effects of media stories of hope and recovery on suicidal ideation and help-seeking attitudes and intentions: systematic review and meta-analysis. The Lancet Public Health. 2022;7:e156–68. https://doi.org/10.1016/S2468-2667(21)00274-7.

14. Niederkrotenthaler T, Sonneck G. Assessing the Impact of Media Guidelines for Reporting on Suicides in Austria: Interrupted time Series Analysis. Aust N Z J Psychiatry. 2007;41:419–28. https://doi.org/10.1080/00048670701266680.

15. Niederkrotenthaler T, Sonneck G, Dervic K, Nader IW, Voracek M, Kapusta ND, et al. Predictors of Suicide and Suicide Attempt in Subway Stations: A Population-based Ecological Study. J Urban Health. 2012;89:339–53. https://doi.org/10.1007/s11524-011-9656-4.

16. Chow S, Men VY, Zaheer R, Schaffer A, Triggs C, Spittal MJ, et al. Suicide on the Toronto Transit Commission subway system in Canada (1998–2021): a time-series analysis. The Lancet Regional Health - Americas. 2024;34:100754. https://doi.org/10.1016/j.lana.2024.100754.

17. Sinyor M, Schaffer A, Redelmeier DA, Kiss A, Nishikawa Y, Cheung AH, et al. Did the suicide barrier work after all? Revisiting the Bloor Viaduct natural experiment and its impact on suicide rates in Toronto. BMJ Open. 2017;7:e015299. https://doi.org/10.1136/bmjopen-2016-015299.

18. Bottomley C, Scott JAG, Isham V. Analysing Interrupted Time Series with a Control. Epidemiologic Methods. 2019;8:20180010. https://doi.org/10.1515/em-2018-0010.
